# Effects of targeted delivery of propionate to the human colon on appetite regulation, body weight maintenance and adiposity in overweight adults

**DOI:** 10.1136/gutjnl-2014-307913

**Published:** 2014-12-10

**Authors:** Edward S Chambers, Alexander Viardot, Arianna Psichas, Douglas J Morrison, Kevin G Murphy, Sagen E K Zac-Varghese, Kenneth MacDougall, Tom Preston, Catriona Tedford, Graham S Finlayson, John E Blundell, Jimmy D Bell, E Louise Thomas, Shahrul Mt-Isa, Deborah Ashby, Glen R Gibson, Sofia Kolida, Waljit S Dhillo, Stephen R Bloom, Wayne Morley, Stuart Clegg, Gary Frost

**Affiliations:** 1 Nutrition and Dietetic Research Group, Section of Investigative Medicine, Imperial College London, 6th Floor Commonwealth Building, Hammersmith Hospital, London, UK; 2 Stable Isotope Biochemistry Laboratory, Scottish Universities Environmental Research Centre, University of Glasgow, Glasgow, UK; 3 Section of Investigative Medicine, Imperial College London, Hammersmith Hospital, London, UK; 4 School of Science, University of the West of Scotland, Hamilton, UK; 5 Institute of Psychological Sciences, University of Leeds, Leeds, UK; 6 Metabolic and Molecular Imaging Research Group, MRC Clinical Science Centre, Imperial College London, Hammersmith Hospital, London, UK; 7 Imperial Clinical Trials Unit, School of Public Health, Imperial College London, London, UK; 8 Department of Food and Nutritional Sciences, University of Reading, Reading, UK; 9 Leatherhead Food Research, Randall's Road Leatherhead, Surrey, UK; 10 Diabetes and Metabolism Division, Garvan Institute of Medical Research, Darlinghurst, NSW 2010, Australia

**Keywords:** NUTRITIONAL SUPPLEMENTATION, OBESITY, GUT HORMONES, APPETITE, COLONIC FERMENTATION

## Abstract

**Objective:**

The colonic microbiota ferment dietary fibres, producing short chain fatty acids. Recent evidence suggests that the short chain fatty acid propionate may play an important role in appetite regulation. We hypothesised that colonic delivery of propionate would increase peptide YY (PYY) and glucagon like peptide-1 (GLP-1) secretion in humans, and reduce energy intake and weight gain in overweight adults.

**Design:**

To investigate whether propionate promotes PYY and GLP-1 secretion, a primary cultured human colonic cell model was developed. To deliver propionate specifically to the colon, we developed a novel inulin-propionate ester. An acute randomised, controlled cross-over study was used to assess the effects of this inulin-propionate ester on energy intake and plasma PYY and GLP-1 concentrations. The long-term effects of inulin-propionate ester on weight gain were subsequently assessed in a randomised, controlled 24-week study involving 60 overweight adults.

**Results:**

Propionate significantly stimulated the release of PYY and GLP-1 from human colonic cells. Acute ingestion of 10 g inulin-propionate ester significantly increased postprandial plasma PYY and GLP-1 and reduced energy intake. Over 24 weeks, 10 g/day inulin-propionate ester supplementation significantly reduced weight gain, intra-abdominal adipose tissue distribution, intrahepatocellular lipid content and prevented the deterioration in insulin sensitivity observed in the inulin-control group.

**Conclusions:**

These data demonstrate for the first time that increasing colonic propionate prevents weight gain in overweight adult humans.

**Trial registration number:**

NCT00750438.

**Significance of this study**

**What is already known about this subject?**

- Increased intake of dietary fibre has been associated with reduced appetite and weight loss.
- The short chain fatty acids (SCFAs) produced by microbial fermentation of dietary fibre in the colon stimulate the release of the anorectic gut hormones peptide YY (PYY) and glucagon like peptide-1 (GLP-1) from rodent enteroendocrine L cells via activation of the G protein coupled free fatty acid receptor (FFAR) 2.
- Of the SCFAs produced by colonic fermentation of dietary fibre, propionate has the highest affinity for FFAR 2.
- Mice receiving a faecal transplant from a donor with a gut microbiota composition that produces elevated levels of propionate in the colon have reduced weight gain and adiposity.

**What are the new findings?**

- Propionate stimulates the release of PYY and GLP-1 from primary cultured human colonic cells.
- This first-in-human study demonstrates that delivery of propionate directly to the colon, acutely increases the release of PYY and GLP-1 and reduces energy intake.
- Long-term colonic propionate delivery prevents body weight gain and reduces intra-abdominal fat accretion in overweight adults.
- Long-term colonic propionate delivery significantly reduces intrahepatocellular lipid content in adults that meet the diagnostic criteria for non-alcoholic fatty liver disease.

**How might it impact on clinical practice in the foreseeable future?**

- Optimising colonic propionate production through selection of propiogenic dietary fibres may represent a novel route to prevent weight gain throughout life and improve public health.

## Introduction

Evidence published over the last 25 years demonstrates that hormonal and neuronal signals from the GI tract play an important role in appetite regulation.^1^ There is increasing evidence that the gut microbiota influences energy regulation and can be a major determinant in the development of obesity.^2^ Recent investigations suggest that diet, the gut microbiota and fat storage can be linked through a molecular mechanism involving short chain fatty acids (SCFAs), the major products of dietary fibre fermentation by the gut microbiome.^3–5^

A major public health challenge is the development of effective strategies that can prevent the increased prevalence in obesity and the reported average 0.3–0.8 kg/year weight gain in adults.^6–9^ Such gradual long-term weight gain can be the result of a small habitual positive energy balance of 50–100 kcal/day.^10^ Interventions that can be safely applied at the population level to reverse this minor energy imbalance and prevent weight gain throughout life would therefore have substantial benefits to public health. Increased intake of dietary fibre has been associated with reduced appetite and weight loss.^11–13^ In particular, evidence suggests that the fermentable component of dietary fibre is critical in mediating these satiating effects.^14^ Feeding rodents a high level of fermentable dietary fibre protects against high-fat, diet-induced increases in body weight and fat mass.^15^ ^16^ There is also evidence that fermentable dietary fibre can suppress appetite and decrease body weight in humans.^17–19^ However, large amounts of dietary fibre (>30 g/day) are required for these beneficial effects, and compliance with high fibre diets is poor, due to GI side effects, which may also explain the inconsistent reports regarding their effects on appetite and body weight.^20^ Targeting the mechanisms by which fermentable dietary fibre suppresses appetite may thus provide a more effective approach to weight control than the use of high fibre diets.

The SCFAs produced by microbial fermentation of dietary fibre in the colon have been shown to stimulate the release of the anorectic gut hormones peptide YY (PYY) and glucagon like peptide-1 (GLP-1) from rodent enteroendocrine L cells.^21–23^ These hormones are released from the gut and are involved in the short-term signal of satiation and satiety to the appetite centres of the brain.^24^ Peripheral administration of PYY_3–36_ or GLP-1 enhances satiety and reduces food intake in animals and man.^25–27^ Recent evidence suggests that SCFAs stimulate GLP-1 release in rodents via stimulation of the G protein coupled free fatty acid receptor (FFAR) 2 on colonic L cells.^21^ Of the SCFAs produced by colonic fermentation of dietary fibre, propionate has the highest affinity for FFAR 2.^28^ ^29^ Furthermore, propionate is an end product of bacterial metabolism, and thus, unlike acetate, does not undergo conversion to other SCFAs.^30^ Intriguingly, Roux-en-Y gastric bypass, which results in weight loss and reduced adiposity, promotes increased levels of propionate in the colon.^5^ In keeping with these findings, a significant negative correlation between adiposity and caecal propionate concentrations has also been reported in germ-free mice receiving faecal transplants from human twin donors discordant for obesity.^4^

Increasing colonic propionate is therefore an attractive target for appetite modulation. However, orally administered SCFAs are unpalatable and are rapidly absorbed in the small intestine where L cells are sparse. Furthermore, supplementing diets with mixed high fibre does not predictably or reliably increase colonic production or circulating levels of propionate in all human populations because of the variability in gut microbial activity.^31^ To overcome the unpalatably high levels of fermentable dietary fibre needed to significantly increase colonic propionate levels, and the unpredictability in the production of the resulting SCFAs, we have developed a novel delivery system targeting the release of gram quantities of propionate in the proximal colon. We hypothesised that propionate would stimulate anorectic gut hormone release from the colon and that targeted delivery of propionate to the colon would decrease appetite and prevent long-term weight gain in humans.

## Methods

### *In vitro* gut hormone secretion experiments

The effect of propionate on PYY and GLP-1 release from human colonic crypts was determined using a modified version of an established method^32^ (see online supplementary material).

### Inulin-propionate ester for colonic delivery of propionate in humans

We developed a novel carrier molecule whereby propionate is chemically bound by an ester bond to inulin, a natural polymer composed mainly of fructose. This inulin-propionate ester was synthesised, as detailed in the online supplementary material. The majority of propionate chemically bound to inulin should only be released when the inulin polymer is fermented by the colonic microbiota, thus providing targeted colonic delivery. Isotope labelling studies were conducted to assess the stability of the molecule through the stomach and small intestine, and to provide information about site and extent of propionate release, as described in the online supplementary information. In addition, the effects of inulin-propionate ester on fermentation profiles and gut microbial populations were studied using an *in vitro* culture system (see online supplementary material).

### Clinical studies

All subjects provided informed, written consent prior to the clinical trial which was approved by the Hammersmith and Queen Charlotte's Research Ethics Committee (08/H0707/99). All studies were carried out in accordance with the Declaration of Helsinki. All clinical trials where registered (Registration No: NCT00750438).

### Investigation of acute supplementation with inulin-propionate ester on appetite regulation

In first-in-human studies, the acute effects of inulin-propionate ester on appetite regulation, hormone release and energy intake were studied in 20 volunteers. The primary outcome was energy intake, and gut hormone release was a secondary outcome. The effects on gastric emptying were examined in 14 volunteers in a separate study. Detailed inclusion and exclusion criteria and methodology for each acute study are described in the online supplementary material.

### Proof-of-principle investigation of the effect of long-term supplementation with inulin-propionate ester on body weight maintenance

We hypothesised that daily intake of inulin-propionate ester over 24 weeks would decrease weight gain in overweight adults. The predefined coprimary outcomes were changes in body weight and food intake. A change in adipose tissue distribution was a secondary outcome. Men and women aged 40–65 years, with a body mass index (BMI) of 25–40 kg/m^2^ were recruited. Potential participants were excluded if they met any of the following criteria: clinically significant illness (including type 1 or type 2 diabetes), medication known to affect appetite and/or body weight, a weight loss of 3 kg or greater in the preceding 2 months, smoking, substance abuse, psychiatric illness and any abnormalities detected on physical examination, electrocardiography or screening blood tests (measurement of complete blood count, electrolytes, fasting glucose, thyroid function and liver function). Women were ineligible if they were pregnant or breast feeding. From an initial 167 persons who responded to letters of invitation, 60 were randomly assigned to either the inulin-control or inulin-propionate ester supplementation group.

#### Study design

The study was conducted using a randomised, double-blind, placebo-controlled, parallel design. Two-day study visits were required at baseline (week 0) and after 24 weeks of dietary supplementation. On the day prior to each study visit, subjects were asked to consume a standard evening meal, to fast overnight from 22:00 and to avoid strenuous physical activity and alcohol. All study visits commenced between 08:00 and 09:00 and were conducted at the National Institute for Health Research (NIHR)/Wellcome Trust Imperial Clinical Research Facility. After all baseline measurements had been taken, subjects were randomly assigned to either the 10 g/day inulin-propionate ester group, or the 10 g/day inulin-control group. Subjects were randomised as described in the online supplementary material. The dietary supplement was supplied to subjects in ready-to-use sachets and they were instructed to mix the contents into their normal diet once a day during the 24-week supplementation period. All subjects were instructed to maintain their usual dietary and physical activity habits during the supplementation period. Self-reported food intake and physical activity were assessed at baseline and after 24 weeks of supplementation (see online supplementary material). Regular communication between subjects and study investigators encouraged good compliance. At week 8 and week 16 of the supplementation period, subjects attend follow-up visits to monitor compliance and adverse events. At week 24, measurements taken at baseline were repeated. Subjects returned all their used and unused sachets to estimate compliance.

#### Body weight and composition

Body weight was measured in all subjects to the nearest 0.1 kg (Tanita BC-418MA) while subjects were wearing light clothing. Body composition was assessed using MRI and MR spectroscopy (MRS), as previously described.^33^ MRI and MRS data could not be collected in 20 subjects, due to metal implants (n=8), claustrophobia (n=9) or technical issues with the scanner (n=3).

#### Appetite regulation, gut hormone release and glucose homoeostasis

A cannula was inserted into an antecubital vein and baseline blood samples collected at −10 min and 0 min to assess plasma concentrations of glucose, insulin, PYY and GLP-1. Following the 0 min sample, subjects were served a standardised breakfast (398 kcal; 71.2 g carbohydrate, 7.9 g fat, 10.3 g protein). At week 24, the breakfast also contained 10 g of inulin-propionate ester or 10 g inulin-control depending on supplementation group. Postprandial blood samples were taken at 15 min, 30 min, 60 min, 90 min, 120 min, 180 min, 240 min and 300 min and collected into heparin-coated tubes containing 0.2 mL of aprotinin (Bayer, UK). GLP-1-like and PYY-like immunoreactivity were measured using established inhouse radioimmunoassay.^34^ ^35^ Insulin and leptin were measured by radioimmunoassay using commercially available kits (Millipore, USA). Plasma glucose was measured using an Abbott Architect ci8200 analyser (Abbott Diagnostics, USA). At 300 min subjects were offered a buffet lunch with food served in excess, and asked to eat until they felt comfortably full. The amount of food was quantified and energy intake calculated. Subjective hunger, satiety and nausea were monitored with the use of 100 mm visual analogue scales (VAS).^36^ Subjects were asked to complete the VAS before each blood sample.

#### Risk factors for cardiovascular disease and diabetes

A fasting blood sample was collected and analysed for levels of triglycerides, total cholesterol, low-density lipoprotein cholesterol, high-density lipoprotein cholesterol, glycosylated haemoglobin (HbA1c), C reactive protein and liver function tests (alanine transaminase, alkaline phosphatase, aspartate transaminase). All analytes were measured by the Department of Chemical Pathology, Imperial College Healthcare National Health Service Trust. Blood pressure and pulse were also measured after subjects had been in a supine position for at least 15 min.

### Statistical analysis

The treatment group size for the acute energy intake study was based on a power calculation, assuming a decrease of 15% in energy intake with a SD of 20% (α=0.05, power=0.85), resulting in an estimated required sample size of 20 subjects. Data from the acute supplementation study suggested a sample size of 50 individuals (25 in each group) was needed for the long-term investigation. Sixty volunteers were therefore recruited to allow for an estimated attrition rate of 15%. χ^2^ tests were performed to compare percentages of subjects in each group who gained ≥3% and ≥5% of their initial weight. For comparison of variables with a single measurement pre supplementation and post supplementation, we calculated the change from baseline at 24 weeks and compared means within groups using paired t-tests. The mean changes between groups for each of these variables were estimated using a multiple linear regression adjusted for its baseline measurement, baseline weight and randomised group. The linear model was also run on logit-transformed variables that were expressed as percentages to ensure their predictive values are within the 0–100% range.^37^ Variables measured multiple (>2) times during the supplementation period (body weight, postprandial glucose, postprandial insulin response, PYY, GLP-1, VAS, side effects assessment) were analysed using multilevel mixed effects models to account for the variability within and between subjects. Area under the curve (AUC), unadjusted for covariates, for postprandial glucose and postprandial insulin response, was also calculated and compared between groups. In the case of data missing at random, the regression techniques described previously were applied after data were imputed using the multiple imputation by chained equations technique to account for random biases of the unobserved covariates. Data are presented as mean±SEM or 95% CI. p Values <0.05 were considered statistically significant.

## Results

### Propionate stimulates PYY and GLP-1 release from human L cells *in vitro*

**Figure 1:**
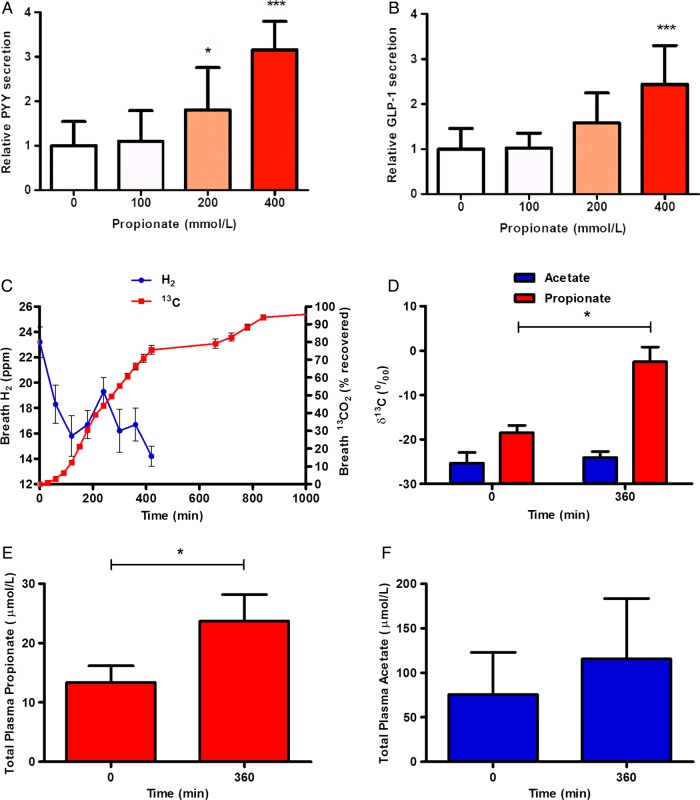
Propionate increases peptide YY (PYY) and glucagon like peptide-1 (GLP-1) release from primary human colonic cells and inulin-propionate ester supplementation delivers propionate to the colon in vivo. Cells isolated from human colonic tissue were incubated with increasing concentrations of propionate. (A) PYY and (B) GLP-1 levels were measured in the supernatants and lysed cells by radioimmunoassay. Percentage gut hormone release per well is expressed relative to the basal release measured (n=4–6). (C) The increase in breath H_2_ at 240 min suggests that >80% of the labelled propionate entered the colon. (D) Plasma acetate and propionate ^13^C enrichment (δ^13^C per mil) at baseline and 360 min. Plasma propionate was significantly more enriched at 360 min whereas no difference was seen in acetate enrichment. Total plasma propionate (E) and acetate (F) concentrations (µmol/L) at baseline and 360 min. Data are presented as mean±SEM, *p<0.05, ***p<0.001.

Propionate significantly stimulated PYY secretion from human colonic cells, with concentrations of 200 mmol/L and 400 mmol/L inducing 1.8-fold and threefold rises above basal secretion, respectively (p<0.05 and p<0.001; figure 1A). Propionate also increased GLP-1 secretion, with 200 mmol/L and 400 mmol/L inducing 1.6-fold and 2.4-fold increases in GLP-1 release, respectively (p<0.001; figure 1B).

### Inulin-propionate ester delivers propionate to the colon

Propionate production from *in vitro* faecal fermentations was significantly higher from inulin-propionate ester compared with inulin, while no significant differences in acetate or butyrate production were observed (see online supplementary figure S1). Following ingestion of 10 g inulin-propionate ester, breath H_2_ started to increase at 180 min and peaked at 240 min post ingestion. A small gradual release of ^13^C was apparent between 0 min and 180 min post ingestion. More than 80% (82.9±2.3%) of the ^13^C recovered in breath over 24 h appeared coincident with and after breath H_2_ onset (figure 1C), suggesting delivery of the majority of the tracer to the colon. It is estimated that 10 g inulin-propionate ester delivered 2.36 g propionate to the colon after accounting for small intestinal losses (0.49 g). The isotopic data show that propionate released from the inulin-propionate ester appeared in the blood and was thus available systemically. Where circulating plasma propionate was detectable, significant increases in propionate ^13^C enrichment and total concentration were measured in peripheral blood 360 min post ingestion compared with baseline (figure 1D, E). No significant differences were observed in plasma acetate enrichment or total concentration (figure 1D, F). We estimate that the addition of 10 g inulin-propionate ester to the diet would lead to a 2.5-fold increase in daily colonic propionate production (see online supplementary material).

### Acute supplementation with inulin-propionate ester increases PYY and GLP-1 secretion and reduces food intake

**Figure 2:**
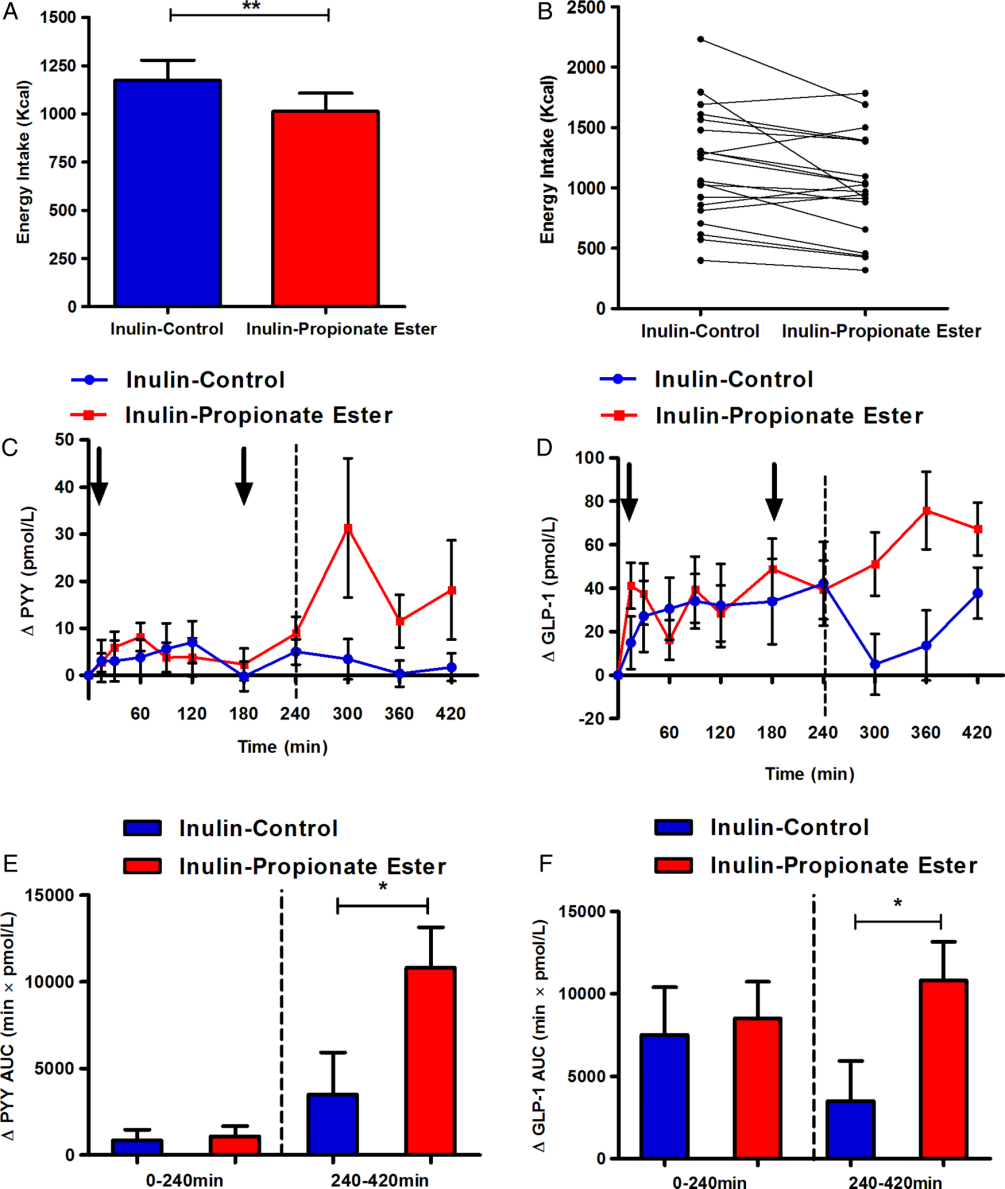
Acute inulin-propionate ester supplementation increases plasma peptide YY (PYY) and glucagon like peptide-1 (GLP-1) levels and reduces energy intake in humans. (A) The mean reduction in energy intake following inulin-control versus inulin-propionate ester. (B) A reduction in energy intake occurred in 16 of the 20 volunteers. (C–F) Plasma gut hormone levels following acute supplementation of inulin-control versus inulin-propionate ester. Arrows indicate standardised meals. Dotted lines signify the time point after which >80% inulin-propionate ester enters the colon as determined by the enrichment of ^13^C in expired air and breath H_2_ methodology (figure 1C). Data are presented as mean±SEM, *p<0.05, **p<0.01. AUC, area under the curve.

Acute supplementation with inulin-propionate ester significantly reduced food intake from 1175 kcal (95% CI 957 to 1392) to 1013 kcal (95% CI 816 to 1210) (figure 2A, B; p<0.01), a mean reduction of 13.8%. It takes an estimated 240 min for the inulin-propionate ester to enter the colon (figure 1C) and compared with inulin-control, inulin-propionate ester significantly increased plasma PYY (ΔAUC_240–420 min_ 429 min×pmol/L (95% CI −543 to 1400) inulin-control vs 3349 min×pmol/L (841 to 5857) inulin-propionate ester, p<0.05) and GLP-1 levels (ΔAUC_240–420 min_ 3495 min×pmol/L (95% CI −1567 to 8558) inulin-control vs 10 801 min×pmol/L (5897 to 15 704) inulin-propionate ester, p<0.05) between 240 min and 420 min. Prior to 240 min there was no significant difference in the concentration of PYY and GLP-1 between the inulin-control and the inulin-propionate ester (figure 2D). Glucose, insulin and leptin levels and subjective ratings of appetite and nausea were not significantly different following acute inulin-propionate ester and inulin-control supplementation (see online supplementary figures S2 and S3). Acute supplementation with inulin-propionate ester did not influence the rate of gastric emptying (see online supplementary information).

### Long-term supplementation with inulin-propionate ester prevents body weight gain, intra-abdominal adipose tissue accretion and reduces intrahepatocellular lipid content in those with non-alcoholic fatty liver disease

Of the 60 volunteers randomised, data were analysed from the 49 participants that completed the 24 week supplementation (table 1). Eleven participants (18%) did not complete the 24 week supplementation and there were no significant differences in attrition between the two groups (see online supplementary figure S4). Baseline and postsupplementation body composition data was collected from 17 participants in the inulin-propionate ester group and 15 participants in the inulin-control group. There was no difference between groups in compliance (95% (95% CI 92% to 98%) inulin-propionate ester vs 94% (95% CI 92% to 97%) inulin-control; p=0.864). Ratings of nausea were not different between supplementation groups (p=0.736), though there were significantly greater ratings of flatulence (p=0.004) in the inulin-control group during the supplementation period compared with the inulin-propionate ester group (see online supplementary table S1).

**Table 1:** Baseline characteristics of subjects and changes in cardiovascular and diabetes risk factors following 24 weeks of inulin-control and inulin-propionate ester supplementation

Variable	Inulin-control (N=24)			Inulin-propionate ester (N=25)			Inulin-propionate Ester−inulin-control	p Value
	Week 0	Week 24	Value	Week 0	Week 24	p Value	Difference (95% CI)	
Sex (% no. of subjects)								
Male	37.5 (9)			40.0 (10)				
Female	62.5 (15)			60.0 (15)				
Race or ethnicity (% no. of subjects)								
White	75.0 (18)			64.0 (16)				
Black	8.3 (2)			16.0 (4)				
Asian	17.7 (4)			20.0 (5)				
Age (years)	53.4±1.5			55.3±1.4				
Weight (kg)	91.0±2.8	91.4±3.0	0.559	88.5±2.9	87.5±3.0	0.062	−1.40 (−3.07 to 0.27)	0.099
Glucose (mmol/L)	5.1±0.1	5.0±0.1	0.624	5.0±0.1	5.0±0.1	0.898	0.05 (−0.21 to 0.32)	0.675
Insulin (µU/mL)	11.5±1.3	10.6±0.7	0.329	9.0±1.0	8.8±0.8	0.702	−0.56 (−2.64 to 1.51)	0.582
HOMA-IR	2.7±0.3	2.3±0.2	0.372	2.0±0.2	2.0±0.2	0.644	0.14 (−0.63 to 0.34)	0.549
HbA1c (mmol/mol)	37.8±0.5	37.1±0.5	0.086	38.2±0.7	37.8±0.7	0.297	0.18 (−0.85 to 1.21)	0.729
Triglycerides (mmol/L)	1.5±0.2	1.6±0.2	0.283	1.5±0.2	1.4±0.2	0.176	−0.29 (−0.68 to 0.11)	0.148
Cholesterol (mmol/L)								
Total	5.3±0.2	5.0±0.2	0.014	5.5±0.2	5.1±0.2	<0.001	−0.10 (−0.39 to 0.19)	0.494
Low-density lipoprotein	3.3±0.2	3.1±0.2	0.132	3.5±0.2	3.2±0.2	<0.001	−0.08 (−0.34 to 0.18)	0.532
High-density lipoprotein	1.4±0.1	1.3±0.1	<0.001	1.4±0.1	1.3±0.1	0.009	0.02 (−0.06 to 0.10)	0.617
Liver function tests (IU/L)								
Alanine transaminase	32.4±3.9	25.9±3.3	0.001	29.5±3.2	23.7±2.2	0.015	−0.28 (−9.30 to 8.75)	0.949
Alkaline phosphatase	78.2±3.4	71.7±3.5	<0.001	74.6±3.7	70.5±3.3	<0.001	1.41 (−2.40 to 5.22)	0.458
Aspartate transaminase	29.8±1.6	28.7±3.0	0.543	28.9±1.3	26.2±1.0	0.007	−1.43 (−7.41 to 4.55)	0.627
Leptin (ng/mL)	31.9±4.0	33.3±4.5	0.690	25.1±3.0	24.1±2.4	0.625	−2.36 (−10.31 to 3.92)	0.551
C reactive protein (mg/L)	3.7±0.5	3.7±0.6	0.818	2.1±0.4	1.8±0.3	0.484	−0.27 (−1.39 to 0.84)	0.622
Blood pressure (mm Hg)								
Systolic	137±2	137±3	0.707	140±3	139±2	0.659	−0.83 (−4.98 to 3.81)	0.688
Diastolic	87±2	85±2	0.143	86±2	84±2	0.101	−0.86 (−4.92 to 3.19)	0.664
Pulse (bpm)	70±2	67±1	0.212	66±2	66±2	0.934	0.63 (−3.36 to 4.61)	0.763

Mean±SEM.

HbA1C, glycosylated haemoglobin; HOMA-IR, homoeostasis model assessment of insulin resistance.

#### Body weight and composition

**Figure 3:**
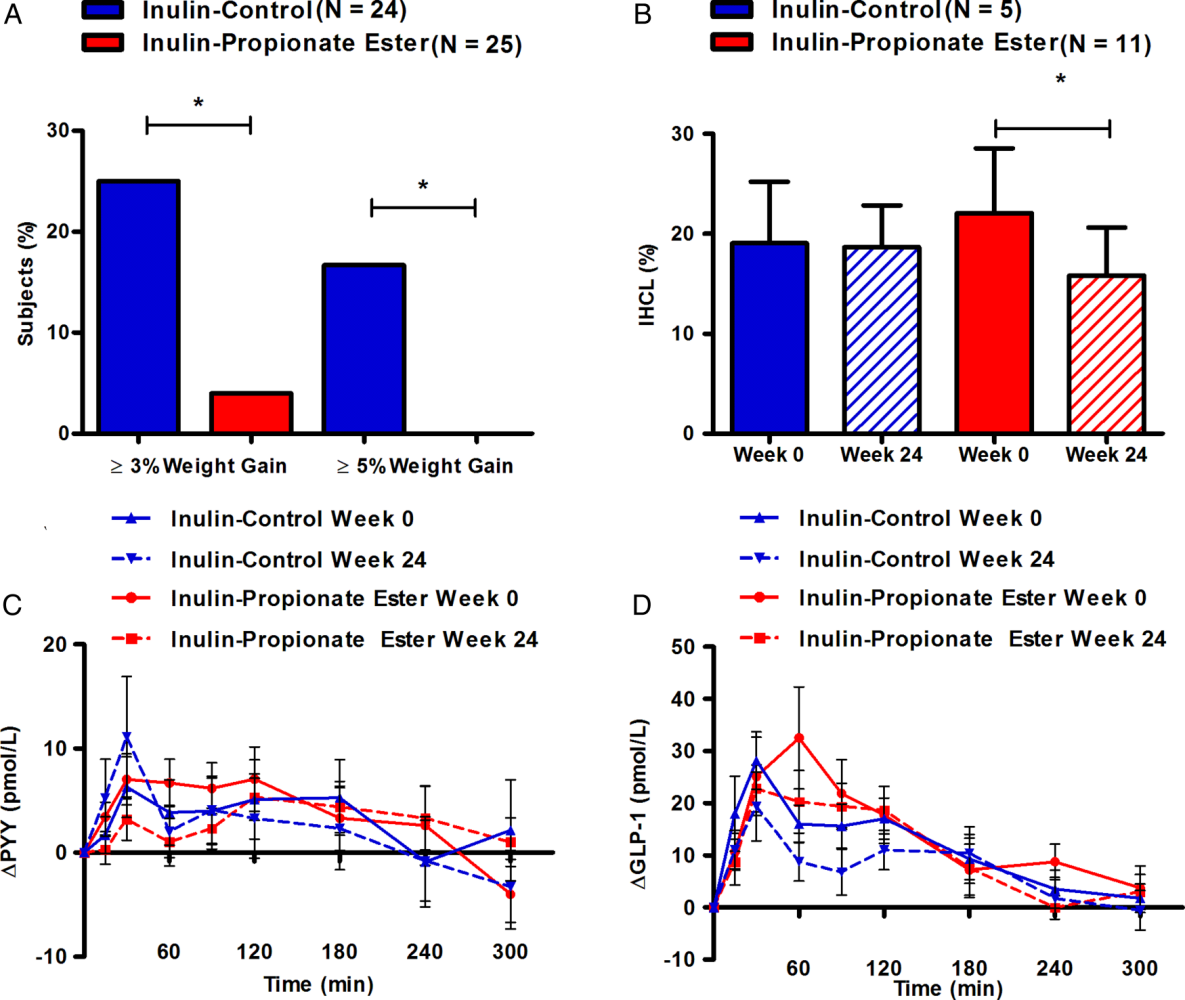
The effect of 24 weeks inulin-control and inulin-propionate ester supplementation on weight gain, liver fat content and gut hormone response. (A) The proportion of subjects who gained 3% or more and 5% or more of their baseline weight at 24 weeks. (B) Intrahepatocellular lipid (IHCL) content at baseline and following 24 weeks of inulin-control and inulin-propionate ester supplementation in subjects with non-alcoholic fatty liver disease (NAFLD). Subjects were identified as having NAFLD on the basis of an IHCL content >5.5% at baseline.^38^ Postprandial plasma (C) peptide YY (PYY) and (D) GLP-1 release at baseline and following 24 weeks of inulin-control and inulin-propionate ester supplementation. Data are presented as mean±SEM, *p<0.05.

**Figure 4:**
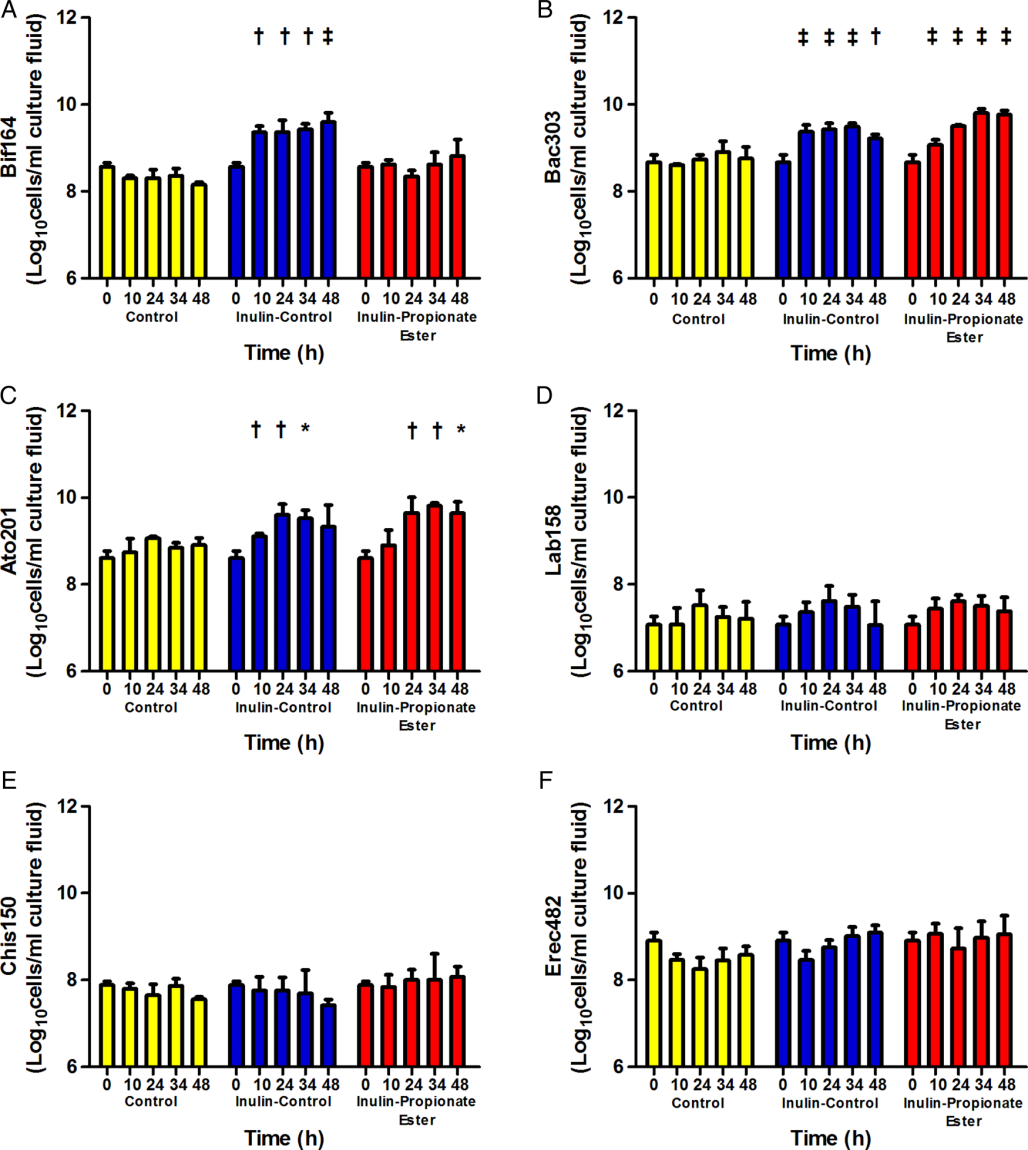
The effect of inulin-propionate ester on the gut microbiota. Bacterial concentrations expressed in Log_10_ cells/mL culture fluid enumerated using fluorescent *in situ* hybridisation (FISH) targeting (A) *Bifidobacterium* spp (Bif164), (B) *Bacteroides/Prevotella* (Bac303), (C) *Atopobium* cluster (Ato291), (D) *Lactobacillus/Enterococcus* (Lab158), (E) *Clostridium histolyticum* (Chis150) and (F) *Eubacterium rectale/Clostridium coccoides* (Erec482) at 0 h, 10 h, 24 h, 34 h and 48 h anaerobic, pH controlled faecal batch culture fermentation with control (no substrate), inulin-control and inulin-propionate ester. Data are presented as mean±SEM (n=3), *<0.05, †<0.001, ‡<0.0001 with respect to the 0 h sample.

Inulin-propionate ester supplementation resulted in beneficial changes in body weight and composition. There was a significant difference in weight gain between groups. One of 25 participants gained ≥3% of their baseline body weight following inulin-propionate ester supplementation (4%), as compared with 6 of 24 participants (25%) in the inulin-control group (figure 3A, p=0.036). Furthermore, none of the participants in the inulin-propionate ester group had substantial weight gain (≥5% baseline weight) compared with 4 of 24 (17%) following inulin-control supplementation (figure 3A; p=0.033). Although the primary aim of the study was to prevent weight gain, it is of interest to note that weight loss after 24 weeks was greater in the propionate ester group, though this effect was not significantly different between groups (0.38 kg (95% CI −0.95 to 1.72) inulin-control vs −1.02 kg (95% CI −2.10 to 0.04) propionate ester, p=0.099). Following the supplementation period, the change in the distribution of intra-abdominal adipose tissue, expressed as a percentage of total adipose tissue content, was significantly lower in the inulin-propionate ester group compared with inulin-control supplementation (table 2; p=0.027). Furthermore, internal adipose tissue (p=0.002) and the ratio of internal adipose tissue: subcutaneous adipose tissue was significantly increased within the inulin-control group (p=0.002), but not in the inulin-propionate ester group. There was no significant change in total adipose tissue content between groups. Within the inulin-propionate ester group there was a trend for reduced intrahepatocellular lipid (IHCL) content post supplementation (p=0.061). However, subjects meeting the baseline diagnostic criteria for non-alcoholic fatty liver disease (NAFLD) (IHCL >5.5%)^38^ had a significant reduction in IHCL content following inulin-propionate ester supplementation (22.1% (95% CI 7.7 to 36.6) to 15.9% (95% CI 5.2 to 26.5), p=0.038, n=11; figure 3B). This effect was not observed in similar subjects within the inulin-control group (19.1% (95% CI 2.0 to 36.1) to 18.7% (95% CI 7.1 to 30.3), p=0.576, n=5; figure 3B). *In vitro* analysis suggested that the protective effects of the inulin-propionate ester on weight gain and adipose tissue distribution were not due to changes in gut bacterial populations compared with inulin-control (see online supplementary material; figure 4A–F).

**Table 2:** Body fat depots at baseline and following 24 weeks of inulin-control and inulin-propionate ester supplementation

AT distribution (% total AT)	Inulin-control (N=15)			Inulin-propionate ester (N=17)			Inulin-propionate ester−inulin-control	p Value
	Week 0	Week 24	p Value	Week 0	Week 24	p Value	Difference (95% CI)	
Subcutaneous AT	81.3±1.9	80.6±1.8	0.002	76.3±1.7	76.0±1.6	0.624	−0.59 (1.69 to 0.50)	0.288
Internal AT	18.7±1.9	19.4±1.8	0.002	23.7±1.7	24.0±1.6	0.624	−0.23 (−1.09 to 0.64)	0.608
Intra-abdominal AT	10.6±1.3	11.1±1.4	<0.001	13.2±1.2	13.1±1.1	0.723	−0.46 (−0.87 to −0.05)	0.027
Abdominal	23.1±0.9	22.7±0.8	0.300	21.9±0.7	21.6±0.7	0.171	−0.23 (−0.88 to 0.42)	0.483
subcutaneous AT								
1H-MRS								
IHCL	7.8±2.9	7.4±2.5	0.708	15.8±5.0	11.5±3.7	0.061	−0.83 (−5.04 to 3.38)	0.699
Soleus IMCL	18.8±2.0	18.8±1.8	0.973	21.8±3.0	24.0±3.7	0.274	2.70 (−1.04 to 6.44)	0.157
Tibialis IMCL	10.0±1.5	10.3±1.1	0.869	9.1±1.0	9.5±0.9	0.701	−0.42 (−2.83 to 1.99)	0.733

Mean±SEM.

IHCL was measured relative to liver water content and IMCL was measured relative to total muscle creatine signal.^33^

AT, adipose tissue; IHCL, intrahepatocellular lipid; IMCL, intramyocellular lipid; MRS, MR spectroscopy.

#### Food intake and gut hormone release following long-term supplementation with inulin-propionate ester

The change in food intake at an *ad libitum* meal following 24 weeks of supplementation was not statistically significant between groups (p=0.972), though long-term inulin-propionate ester intake showed a trend towards a lower food intake by 8.7% from 836 kcal (95% CI 724 to 948) to 763 kcal (95% CI 654 to 872) (p=0.100). Inulin-control supplementation reduced food intake from 678 kcal (95% CI 535 to 820) to 645 kcal (95% CI 514 to 776) (p=0.197), a mean reduction of 4.0%. Subjective ratings of appetite were significantly reduced within the inulin-propionate ester group following the supplementation period, while there were no differences in ratings of nausea (see online supplementary figure S5). Interestingly, there were no significant differences in postprandial PYY (difference −1.48%; 95% CI −6.29% to 3.33%; figure 3C; p=0.546) or GLP-1 secretion (difference 3.69%; 95% CI −4.48% to 12.33%; figure 3D, p=0.361).

#### Glucose homoeostasis

Multilevel mixed effects models found no differences in postprandial glucose (p=0.350) or insulin response (p=0.924) between supplementation groups. However, the change in postprandial glucose AUC to the standardised breakfast at week 24 was significantly different between groups (see online supplementary figure S6; p=0.037). Glycaemic response significantly deteriorated following inulin-control supplementation (AUC_0–300 min_ 1600 min×mmol/L (95% CI 1495 to 1705) to 1691 min×mmol/L (95% CI 1580 to 1802), p=0.010) but remained unchanged following inulin-propionate ester supplementation (AUC_0–300 min_ 1630 min×mmol/L (95% CI 1534 to 1727) to 1630 min×mmol/L (95% CI 1543 to 1717), p=0.993), in keeping with the differences in weight gain and body fat distribution observed within the groups. Insulin AUC was not significantly different following inulin-propionate ester (AUC_0–300 min_ 7685 min×µU/mL (95% CI 5858 to 9511) to 7969 min×µU/mL (95% CI 5955 to 9982), p=0.612) or inulin-control supplementation (AUC_0–300 min_ 9285 min×µU/mL (95% CI 7454 to 11 115) to 8781 min×µU/mL (95% CI 7265 to 10 297), p=0.464) and the change in insulin AUC was not significantly different between groups (see online supplementary figure S7; p=0.372), suggesting that the difference in glycaemic response reflects a difference in insulin sensitivity.

#### Risk factors for cardiovascular disease and diabetes

Inulin-propionate ester and inulin-control supplementation significantly reduced circulating levels of total cholesterol, high-density lipoprotein, alanine transaminase and alkaline phosphatase (table 1). Significant reductions in low-density lipoprotein (p<0.001) and aspartate transaminase (p=0.007) were only observed within the propionate ester group.

## Discussion

The GI tract is an important organ in the short-term control of appetite.^1^ ^24^ The production of SCFAs by microbial fermentation of dietary fibre has been linked to positive physiological effects, including improvements in body weight, adiposity and glucose metabolism.^3^ Our data demonstrate that the SCFA propionate stimulates the release of the anorectic gut hormones PYY and GLP-1 from human colonic cells *in vitro*, supporting observations made in animal models.^21^ To increase colonic propionate production *in vivo*, we designed and synthesised a novel inulin-propionate ester, whereby propionate is conjugated by an ester linkage to the carrier molecule inulin. Stable isotope methodology revealed that >80% of the propionate load from the inulin-propionate ester is released in the colon coincident with or after a rise in breath H_2_. This would suggest that only a relatively small amount of the esterified propionate is released and absorbed in the small intestine. We have estimated that 10 g inulin-propionate ester ingestion leads to a 2.5-fold increase in daily colonic propionate production, a level very difficult to achieve through feeding a mixed fermentable fibre diet.^20^ We also demonstrated that ingestion of the inulin-propionate ester increases plasma propionate levels.

We subsequently, in the first-in-human studies, demonstrated that increased delivery of propionate to the colon acutely modulates gut hormone release and reduces food intake in healthy subjects. The inulin-propionate ester did not suppress subjective appetite responses, but significantly reduced meal size, consistent with the action of a physiological satiation signal. We observed a significantly greater postprandial release of PYY and GLP-1 when a mixed calorie breakfast contained 10 g inulin-propionate ester compared with 10 g inulin-control. It has been previously shown that a sustained increase in circulating PYY and GLP-1 can influence appetite-regulating circuits of the brain and inhibit food intake.^25^ ^27^ In this study, the rise in PYY and GLP-1 was apparent between 240 min and 420 min following oral administration of the inulin-propionate ester, and reached levels similar to those observed following a 1000 kcal meal.^39^ Such a rise did not occur following ingestion of the inulin-control, suggesting that it is a specific effect of the inulin-propionate ester rather than the standardised 356 kcal lunch provided in both trials. This would suggest that compared with the inulin-propionate ester, a 10 g dose of inulin-control does not raise colonic SCFA to a sufficient concentration to stimulate gut hormone release.^40^ Recent evidence suggests the colonic microbiota adapt rapidly to a change in substrate availability.^41^ Data from batch culture experiments demonstrated that inulin-control and inulin-propionate ester stimulate changes to the gut microbiota, although only inulin-control had a selective effect on *Bifidobacterium*. This would suggest that the observed short-term effects on appetite regulation were independent of alterations to gut microbial composition.

Longitudinal studies demonstrate that adults gain weight gradually through middle age, with an average yearly weight gain of 0.3–0.8 kg.^6–9^ This accumulation of weight would result from a small daily positive energy balance of around 50–100 kcal.^10^ The 14% (162 kcal) reduction in food intake observed following acute administration of 10 g/day inulin-propionate ester would have a significant impact on weight gain and health if sustained over the long term.^42^ We therefore investigated if long-term elevation of colonic propionate would prevent weight gain by conducting a 24 week randomised controlled trial of inulin-propionate ester supplementation in overweight middle-aged adults. We demonstrated lower weight gain in the inulin-propionate ester group, with significantly fewer volunteers gaining ≥3% or ≥5% body weight. This was coupled with a reduced gain in intra-abdominal adipose tissue compared with the inulin-control group and prevention of the deterioration of postprandial glucose response. Furthermore, long-term elevations in colonic propionate production reduced IHCL content in subjects meeting the diagnostic criteria for NAFLD. A reduction in IHCL is a reproducible finding in rodents fed a high level of fermentable dietary fibre,^16^ ^43^ however the mechanism behind this is not well understood. Intra-abdominal adipose tissue and NAFLD are regarded as major risk factors in the development of insulin resistance and type 2 diabetes.^44^

Interestingly, in this long-term study we were unable to detect any change in PYY or GLP-1 release following ingestion of the inulin-propionate ester compared with inulin-control, in contrast to our acute administration studies. This suggests that there may be a desensitisation of the FFAR2/3 receptor response over time and that the beneficial effects of long-term inulin-propionate ester supplementation may not be mediated by PYY and GLP-1. However, subjective ratings of postprandial appetite were significantly reduced within the inulin-propionate ester group and we observed a trend towards a significant decrease in food intake of 8.7% (73 kcal), suggesting that propionate may influence appetite and energy intake via mechanisms unrelated to PYY or GLP-1 release. It has been demonstrated that propionate can stimulate leptin release through activation of FFAR2 on adipocytes,^45^ although we did not observe any changes in circulating leptin concentrations following acute or long-term supplementation with inulin-propionate ester. Recent reports suggest that propionate could also have a positive effect on energy balance and body weight independent of energy intake. An investigation observed weight loss in germ-free mice transplanted with microbiota from animals which had undergone gastric bypass surgery. The reduced body weight was associated with increased microbial production of propionate, but no differences in energy intake were observed.^5^ In addition, when the intestines of germ-free mice are transplanted with microbiota from an obese or lean human twin it was found that animals receiving the transplant from the lean twin donor developed decreased lower body mass and adiposity level compared with those receiving the transplant from the obese twin, despite comparable energy intake.^4^ The inhibition of adipose tissue accumulation observed in the lean twin transplanted mice was associated with greater amounts of propionate produced by the gut microbiota. The outcome of these investigations could be attributed to the observation that propionate promotes sympathetic activity via FFAR3, resulting in elevated energy expenditure.^46^ Furthermore, SCFA activation of FFAR2 has been shown to reduce the sensitivity of murine adipocytes to insulin, leading to reduced lipid clearance by adipocytes and to increased energy expenditure, with preferential oxidation of lipid.^47^ Propionate has also been shown to stimulate a gut–brain circuit via FFAR3 in the portal vein wall, leading to the induction of intestinal gluconeogenesis (IGN) gene expression.^48^ The authors suggest that the glucose released by IGN is detected by a portal vein glucose sensor that transmits its signal to the brain by the peripheral nervous system to promote beneficial effects on energy homoeostasis. Using a rodent-model it was found that upregulation of IGN by propionate reduced body weight gain and adiposity independent of food intake. These reports indicate that propionate can contribute to energy homoeostasis through effects on numerous cellular metabolic pathways and receptor-mediated mechanisms and provide a potential explanation for the differences in body weight gain and adiposity observed between supplementation groups in the long-term study. Additional investigations are therefore warranted to clarify the effects of long-term supplementation with the inulin-propionate ester on energy expenditure and the metabolic and neural pathways that regulate substrate oxidation. Given that acutely elevating colonic propionate increases plasma PYY and GLP-1 levels and inhibits energy intake in healthy subjects, and that this effect on gut hormone release appears to be lost following long-term supplementation while a reduction in body weight gain is maintained, the short-term and long-term effects of colonic propionate may have divergent underlying biological mechanisms.

A possible limitation of our study design would be the choice of inulin as a control for the inulin-propionate ester. Inulin was used as a control to specifically account for any effects that may derive from colonic fermentation of inulin itself, rather than the release of the esterified propionate. As our *in vitro* faecal fermentation data demonstrates, the levels of propionate produced by the inulin-control are relatively small compared with those produced by the inulin-propionate ester, but the production of acetate and butyrate are comparable. Previous studies have shown that much larger doses of inulin-type fructans (>30 g/day) are required to modulate gut hormone release and appetite regulation than used as a control in the present design.^40^ ^49^ It is therefore unlikely that the 10 g/day dose of inulin used as a control is masking any effects of the inulin-propionate ester on our primary and secondary outcome measures. Nevertheless, the SCFA production from 10 g/day inulin may be sufficient to explain some of the significant long-term changes observed within the inulin-control group, particularly the reductions in fasting cholesterol.^50^

In summary, these studies provide the first direct evidence that colonic propionate can acutely reduce energy intake and prevent long-term weight gain in humans. The present results support a role specifically for colonic propionate in weight management and may provide a molecular explanation of recent data that have observed changes in the gut microbiome and associated SCFA production profiles in weight loss. In humans, the beneficial actions of propionate appear to be mediated by different mechanisms in the short term compared with the long term, which warrants further study. Optimum delivery of propionate to the colon through selection of propiogenic components of the diet may represent a novel route to improve weight management at the population level.

## Supplementary Material

Web supplement
