# Supplementary material for: Effects of targeted delivery of propionate to the human colon on appetite regulation, body weight maintenance and adiposity in overweight adults
Source: Gut. 2014 Dec 10;64(11):1744–54. doi: 10.1136/gutjnl-2014-307913 (PMC4680171; doi:10.1136/gutjnl-2014-307913)
Supplement: Web supplement [file gutjnl-2014-307913-s1.pdf]

**SUPPLEMENTARY MATERIAL**

**CONTENTS**


| | **PAGE** |
|---|---|
| **SUPPLEMENTARY METHODS** | |
| **Isolation of human colonic cells** | 2 |
| **In vitro gut hormone secretion experiments** | 2 |
| **Production of inulin-propionate ester** | 3 |
| ***In vitro* fermentation profiles of inulin-propionate ester and inulin-control** | 4 |
| **Acute intervention with inulin-propionate ester study subjects** | 5 |
| **Colonic delivery investigation** | 5 |
| **Gut hormone and energy intake investigation** | 6 |
| **Gastric emptying investigation** | 6 |
| **Long-term supplementation with inulin-propionate ester** | 7 |
| **Fluorescent in situ hybridisation to assess gut microbial changes in response to inulin propionate ester and control** | 7 |
| **Statistical analysis** | 9 |
| **SUPPLEMENTARY RESULTS** | 10 |
| **SUPPLEMENTARY FIGURES** | 12 |
| **SUPPLEMENTARY TABLES** | 20 |
| **REFERENCES** | 22 |

## SUPPLEMENTARY METHODS

### Isolation of human colonic cells

Colonic biopsies were obtained from patients undergoing diagnostic colonoscopy at Hammersmith Hospital. Biopsies were usually obtained from the right side of the colon. All subjects provided informed, written consent prior to the study, approval for which was granted by the Hammersmith and Queen Charlotte's Research Ethics Committee (Registration No: 2000/5795).

The colonic tissue was prepared as described previously [1 2]. Briefly, the tissue was digested with 0.4mg/ml collagenase XI (Sigma, UK) in Dulbecco's Modified Eagle Medium (DMEM) at 37$^{o}$C. The resulting cell suspensions were centrifuged for 5 minutes at 500 × *g* and the pellets re-suspended in DMEM (supplemented with 10% foetal calf serum (FCS) and 1% penicillin/streptomycin). The digestion process was repeated three times and the combined cell suspensions plated onto 24-well, 1% Matrigel-coated plates and incubated at 37$^{o}$C in an atmosphere of 5% $CO_2$.

### *In vitro* gut hormone secretion experiments

Secretion experiments were carried out 18-24 hours after the colonic cells were plated. Cells were washed twice with secretion buffer (4.5mM KCl, 138mM NaCl, 4.2mM $NaHCO_3$, 1.2mM $NaH_2PO_4$, 2.6mM $CaCl_2$, 1.2mM $MgCl_2$ and 10mM HEPES, adjusted to pH 7.4 with NaOH) containing 0.1% bovine serum albumin (BSA) [1] and incubated with sodium propionate (Sigma, UK) for 2 hours at 37$^{o}$C in an atmosphere of 5% $CO_2$. Concentrations of 200 and 400 mmol/L were used as estimates suggest that the concentration of SCFA in the human colon is approximately 150 mmol/L and modelling data from *in vitro* systems demonstrate that these levels will more than double with a high fermentable carbohydrate diet [3 4]. In addition, these concentrations are in line with those previously shown to stimulate PYY release from the rodent colon (500 mmol/L) [5]. Following incubation, cell supernatants were centrifuged and the plates treated with lysis buffer and freeze-thawed. Percentage gut hormone release was calculated for each well and normalised to basal secretion [1]. Cell health was confirmed using a CytoScan™ lactate dehydrogenase assay (G-Biosciences, USA). PYY and GLP-1 data was obtained from separate cell cultures.

**Production of inulin-propionate ester**

The inulin-propionate ester was synthesized by reacting inulin with propionic anhydride (0.8 L/Kg inulin) in water whilst maintaining pH between 8 - 8·5 and the reaction temperature < 20°C. Excess unreacted propionate was removed by filtration twice through activated carbon columns whilst the mixture was maintained at pH 2. Finally, the product was recovered as a fine amorphous crystalline product through spray drying. A $^{13}C$ stable isotope labelled variant of the propionate ester was synthesised in an identical fashion to produce $^{13}C$-propionate ester with (1-$^{13}C_1$)-propionate bound to inulin.

To determine the amount of free propionate in the final product, 100 mg of ester product was dissolved in 2 ml water containing 10 mM butyric acid as the internal standard (IS). To quantify free propionate, 200 µl of the solution was treated with 100 µl of concentrated orthophosphoric acid followed immediately by ether extraction (1 ml). To quantify total propionate (free + bound), another 200 µl of the solution was treated with 100 µl of concentrated orthophosphoric acid and heated at 80°C for 1 hour before being extracted with 1 ml ether. Propionate and butyrate in the ether extracts were quantified by gas chromatography with flame ionisation detection [6]. Propionate yield was calculated relative to the IS and the amount of free propionate calculated by the ratio (free/total) × 100 (%). The degree of esterification ($d_e$) was also calculated using this analysis by using the yield of bound propionate (total – free) per gram of ester to compute moles of propionate yielded per mole of propionate ester.

An inulin-propionate ester was produced with a degree of esterification of 0.74 ± 0.02, meaning on average that 24.6 ± 0.67% (0.74 out of maximum of 3 per monosaccharide unit) of all hydroxyl groups were replaced by an ester group. The level of free propionate was 2.57 ± 0.26 % of the total propionate available from the molecule, demonstrating that more than 97% of propionate was chemically bound to the inulin polymer. Less than 1% of the bound propionate is released when the inulin-propionate ester is solubilised in acid (pH 1-2), a similar low pH to the environment of the stomach. Heating to >80ºC is required in order to release the propionate from the ester in this preparation.

***In vitro* fermentation profiles of inulin-propionate ester and inulin-control**

Faecal samples were collected from three healthy volunteers (who had no history of gastrointestinal complaints and were antibiotic free for >6 months prior to faecal collection) and prepared separately in triplicate for each substrate. Faecal fermentation systems consisted of the fermentation medium (2.25g tryptone in 450 ml of distilled water), 112.5 µl of micromineral solution (13.2 g of $CaCl_2$, 10.0 g of $MnCl_2$, 1.0 g $CoCl_2$, $FeCl_3$ made up to 100ml with distilled water), 225 ml of macromineral solution (2.85 g $Na_2HPO_4$, 3.1 g $KH2PO_4$, 0.3 g $MgSO_4$ made up to 500 ml with distilled water), 225 ml of buffer solution (2 g $NH_4HCO_3$, 17.5 g $NaHCO_3$ made up to 500 ml with distilled water) and 1 ml of 0.1% (w/v) resazurin solution (a redox indicator). This medium was adjusted to pH 7 using 6M HCl, after which it was sterilized by boiling for 5 min. Reducing solution prepared on the day of fermentation (312.5 mg cysteine hydrochloride, 2 ml 1M NaOH, 312.5 mg sodium sulfide, and 47.5 ml distilled water) was added at 0.5 ml per 10 ml of medium after the solution was cooled to 37°C under oxygen-free nitrogen (OFN) until anaerobic conditions were achieved as indicated by a colour change from pale indigo to colourless. A 32% faecal slurry was prepared for each subject in 66.6 mM phosphate buffer (pH=7), homogenized in a household blender for 2 min and strained through a nylon stocking. The final *in vitro* system containing 0.5 ml of the slurry was added to 4.2 ml of the pre-reduced fermentation medium in 10 ml autoclaved fermentation bottles. Each batch culture consisted of 4.9 ml of the above slurry mixture to which 100 mg of inulin-propionate ester or inulin-control was added. At 0 and 24 h, 800 µL of the aqueous volume was removed from each vial. For SCFA extraction, 100 µL of internal standard (IS; 2-ethylbutyrate, 73.8 mM) and 25 µL concentrated orthophosphoric acid was added to 225 µL of sample and thoroughly mixed and extracted with 3 x 1 mL of ether. The ether aliquots were pooled and a sub-sample transferred to a clean vial for analysis. Samples were analysed by GC-FID (Trace GC, ThermoFisher, UK) using a ZB-WAX column (15 m × 0.53 mm × 0.25 um; Phenomenex, Cheshire UK). The GC operating parameters were nitrogen carrier gas (1.89 ml/min) and GC oven parameters starting at an initial temp of 80°C, ramp temp (10°C /min) to 210°C with splitless injection. The concentration of acetate, propionate and butyrate calculated using the area ratio to the IS and the calibrated response factor of each SCFA to the IS as determined by a gravimetrically prepared external standard. The concentration (production) of SCFAs and molar ratios were calculated.

**Acute intervention with inulin-propionate ester**

Nine healthy subjects (8 males and 1 female) were recruited for the colonic delivery investigation. The mean (± SEM) age, weight and body mass index (BMI) were 32 ± 4 years, 75.0 ± 4.0 kg and 25.0 ± 1.3 kg/$m^2$, respectively. Twenty healthy subjects (15 males and 5 females) were recruited for the acute food intake investigation. The mean (± SEM) age, weight and BMI were 31 ± 2 years, 75.0 ± 3.0 kg and 25.4 ± 0.8 kg/$m^2$, respectively. Fourteen healthy subjects (8 males and 6 females) were recruited for the gastric emptying investigation. The mean (± SEM) age, weight and BMI were 32 ± 4 years, 69.4 ± 3.5 kg and 24.0 ± 0.9 kg/$m^2$, respectively. The inclusion criteria for these investigations were a BMI of 20 to 35 kg/$m^2$ and 21 to 65 years of age. The exclusion criteria were smoking, substance abuse, pregnancy, use of medications (except for oral contraceptives), a change in body weight >3 kg in the previous 2 months, medical or psychiatric illness, and any abnormalities detected on physical examination, electrocardiography, or screening blood tests (measurement of complete blood count, electrolytes, fasting glucose, thyroid function and liver function).

**Colonic delivery investigation**

This investigation compared the appearance of $^{13}C$ in breath $CO_2$ with the appearance of breath $H_2$, a methodology previously used to investigate gut transit times [7]. Subjects arrived at Hammersmith Hospital at 08:30 and were served a standardized breakfast (533 kcal; 78 g carbohydrate (CHO), 17 g fat, 18 g protein) containing 10g of propionate ester and 100mg $^{13}C$ labelled inulin propionate ester (containing ~30 mg of bound (1-$^{13}C_1$)-propionate). Breath $H_2$ was collected and measured in real-time using a handheld $H_2$ monitor (Bedfont Scientific Ltd, Kent UK). Breath $CO_2$ was collected serially over 24 h (excluding the sleep period) by exhaling alveolar breath through a straw into Exetainers (Labco, Buckinghamshire, UK). $^{13}CO_2$ enrichment was determined by isotope ratio mass spectrometry (IRMS). Breath $H_2$ was expressed as parts per million (ppm) and $^{13}CO_2$ as ppm xs, defined as ppm $^{13}C$ enrichment above baseline samples collected before isotope ingestion. Cumulative $^{13}CO_2$ excretion was also calculated. Plasma was collected at -15, 0 and 360 min, and urine over 24 h for analysis of $^{13}C$ propionate enrichment by GC-combustion-IRMS (GC-C-IRMS) [6]. Acetate $^{13}C$ enrichment was also measured in the same analysis as a control for dietary influence on SCFA $^{13}C$ enrichment and for evidence of inter-conversion between propionate and acetate. Although our experimental methodology was not designed to explore gluconeogenesis, $^{13}C$ enrichment was measured in plasma glucose and plasma alanine by LC-IRMS [8] and GC-C-IRMS [9], respectively. Enrichment in plasma $^{13}C$ glucose would occur through gluconeogenesis from propionate directly but alanine would indicate the enrichment in the triose pool, with which it

comes rapidly into isotopic equilibrium [10]. Data were expressed as $\delta^{13}C$ (per mil or ‰) which represents the change in the measured ratio in parts per thousand from the internationally accepted standard carbon Vienna Pee Dee Belemnite (VPDB). Isotopic enrichment was expressed as $\delta^{13}C$ (‰). SCFA concentrations (µmol/L) were measured relative to an internal standard (3-methyl valerate).

**Energy intake and gut hormone investigation**

The study was performed in a randomized, double-blind, crossover manner, with each subject studied on two occasions > 7 days apart. Subjects refrained from alcohol and strenuous exercise for the 24 hours prior to each study day and consumed an identical meal between 19:00 and 20:00 the evening before. Subjects then fasted overnight and arrived at Hammersmith Hospital at 08:30 on each study day. A cannula was inserted into a forearm vein and baseline blood samples were collected at -10 and 0 min. Following the 0 min sample, subjects were served a standardized breakfast (398 kcal; 71 g CHO, 8 g fat, 10 g protein) containing either 10 g of inulin-propionate ester, or 10 g inulin control. At 180 min a standardized lunch (356 kcal; 34 g CHO, 12 g fat, 28 g protein) was provided, and at 420 min subjects were offered a buffet dinner with food served in excess and asked to eat until they felt comfortably full. The amount of food was quantified and energy intake calculated. Postprandial blood samples were taken at 15, 30, 60, 90, 120, 180, 240, 300, 360 and 420 min and collected into heparin-coated tubes containing 0.2 ml of aprotonin (Bayer, UK). Plasma was separated immediately by centrifugation at 4°C and then stored at -70°C until analyzed. Subjective hunger, satiety, and nausea were monitored with the use of 100 mm visual analog scales (VAS) [11]. Subjects were asked to complete the VAS before each blood sample.

**Gastric emptying investigation**

The study was performed in a randomized, double-blind, crossover manner, with each subject studied on two occasions > 7 days apart. Subjects refrained from alcohol and strenuous exercise for the 24 hours prior to each study day and asked to consume a standard meal between 19:00 and 20:00 the evening before. On the study days, subjects attended having fasted overnight. Following baseline blood samples, subjects were given 10 g inulin-propionate ester or 10 g inulin control in a standardized breakfast (641 kcal; 113 g CHO, 15 g fat, 16 g protein). At 300 min volunteers were served a standard lunch (354 kcal; 47 g CHO, 12 g fat, 12 g protein) together with 100 mg $^{13}C$-octanoic acid [12]. Breath $CO_2$ was collected serially for 480 min after the standard lunch by exhaling alveolar breath through a straw into Exetainers (Labco, Buckinghamshire, UK). $^{13}CO_2$ enrichment

was determined by isotope ratio mass spectrometry (IRMS). The time to 50% AUC excretion of $^{13}C$ in breath ($T_{1/2}$) was calculated as a proxy for gastric emptying rate.

## Long-term supplementation with inulin-propionate ester

*Randomisation*

Four strata were defined according to sex (male, female) and BMI (<30.0 kg/m$^2$, ≥30.0 kg/m$^2$) and randomisation sequences for each stratum were generated using the random number generator using Stata (version 11.0). The two supplementation groups were labelled 'A' and 'B' by DJM who held the key to the allocations for the duration of the trial. Participants were randomised to either 'A' or 'B' by the trial statistician (SMI) according to the randomisation sequence and assigned a randomisation number. Throughout the trial, none of the participants or investigators involved in the trial had access to complete information on the randomisation allocations.

*Self-reported assessment of energy intake and physical activity*

Participants were asked to record energy intake by 3-day food diary at baseline and during the last week of the 24 week supplementation period. Food diaries were analysed using Dietplan6 (Forestfield Software, West Sussex, UK). Physical activity was measured at baseline and at week 24 using the short self-administered format of the International Physical Activity Questionnaire (IPAQ)[13]. IPAQ measures the frequency and duration of any walking and other moderate-to-vigorous intensity physical activity undertaken for more than 10-continuous minutes across all contexts (e.g. work, home and leisure) over a 7-day period. IPAQ calculates metabolic equivalents (MET-h/week), derived by assigning standardised MET values for walking, moderate-intensity and vigorous-intensity of 3.3, 4.0 and 8.0, respectively.

## Fluorescent in situ hybridisation to assess gut microbial changes in response to inulin-propionate ester and control

The effects of inulin-propionate ester on gut microbial populations were studied using an *in vitro* culture system. Faecal samples were obtained from three healthy human volunteers (two males; age 30 – 50 years; BMI 25-31 kg/m$^2$). Volunteers were excluded if they suffered from any gastrointestinal disorder (e.g. ulcerative colitis, Crohn's disease, irritable bowel syndrome, peptic

ulcers and cancer) and/or had taken antibiotics in the six months preceding sample donation. Samples were kept under anaerobic conditions (10% $H_2$, 10% $CO_2$ and 80% $N_2$) and used within a maximum of 30 min after voiding. Faecal samples were diluted 1/10 w/w in anaerobic phosphate buffered saline (0.1 mol/l phosphate buffer solution, pH 7.4) and homogenised in a stomacher (Stomacher 400, Seward, West Sussex, UK) for 2 min at normal speed.

*Batch culture fermentations*

Sterile anaerobic batch culture fermenters (150ml working volume) were set up in parallel and aseptically filled with 135ml pre-reduced, sterile basal culture medium (peptone water 2 g/l (Oxoid), yeast extract 2 g/l (Oxoid, Basingstoke, UK), NaCl 0.1 g/l, $K_2HPO_4$ 0.04 g/l, $KH_2PO_4$ 0.04 g/l, $MgSO_4.7H_2O$ 0.01 g/l, $CaCl_2.6H_2O$ 0.01, $NaHCO_3$ 2 g/l, Tween 80 2 ml (BDH, Poole, UK), haemin 0.05 g/l, vitamin $K_1$ 10 μl, cysteine.HCl 0.5 g/l, bile salts 0.5 g/l). The fermenters were gassed overnight with $O_2$ free $N_2$ at a rate of 15mL/min. Inulin-propionate ester and inulin control substrates (1/10 w/v) were added to their respective fermenters just prior to the addition of the faecal slurry. A substrate-free vessel was set up for each volunteer as negative controls. Cultures were continuously stirred and kept at 37°C by means of a circulating water bath. Culture pH was kept between 9.7 and 6.9 using automated pH controllers (Fermac 260, Electrolab, Tewkesbury, UK). Each vessel was inoculated with 15 ml fresh fecal slurry (1/10 w/w). Batch fermentations were ran for 48 h and 6 ml samples were obtained from each vessel at 0, 10, 24, 34 and 48 h for microbial enumeration by fluorescent *in situ* hybridisation (FISH) analysis. Three replicate fermentations were set up, each inoculated with one of three different human fecal samples.

*Bacterial enumeration*

Fluorescent in situ hybridization targeting *Bifidobacterium* spp. (Bif164), *Bacteroides/ Prevotella* (Bac303), *Lactobacillus/ Enterococcus* (Erec482), *Clostridium histolyticum* (Chis150), *Atopobium* cluster (Ato291) and *Eubacterium rectale/ Clostridium coccoides* (Erec482), was used as described by Sarbini et al (2011). Cells were visualized by fluorescent microscopy (Eclipse 400, Nikon, Surrey, UK) using the Fluor 100 lens. For each sample, 15 different fields of view were enumerated.

**Statistical analysis**

One-way ANOVA was performed to determine differences in *in vitro* secreted gut hormone levels. Differences in energy intake and area under the curve (AUC) for plasma hormone levels and gastric emptying between trials were assessed using Paired Student's *t* test. Two-way (trial×time) repeated measures ANOVA was performed to determine differences in plasma hormone levels, VAS, breath hydrogen, and bacterial group populations. Significant effects were followed up by Tukey's *post hoc* comparisons. Data are presented as means ± SEM or ± 95% Confidence Interval [CI]. P values < 0.05 were considered statistically significant.

## SUPPLEMENTARY RESULTS

### Colonic delivery investigation

*$^{13}$C enrichment in plasma glucose and plasma alanine*

Enrichments in plasma $\delta^{13}$C glucose and $\delta^{13}$C alanine were 1.5 ± 1.7 per mil (p=0.27) and 0.9 ± 0.8 per mil (p=0.11) at 360 min compared with baseline, respectively.

*Estimation of daily colonic short chain fatty acid production from inulin-propionate ester*

Data from sudden death victims suggests that the average propionate pool size in the proximal large intestine is 4.5 mmol and 10 g of inulin-propionate ester releases an additional 15.0 mmol and 36.2 mmol from inulin fermentation and bound propionate respectively [3]. Using stoichiometric equations for hexose fermentation by intestinal bacteria [14], we calculate that a daily dietary intake of 15 g non-starch polysaccharide yields a daily production of 94.1, 34.5 and 28.2 mmol for acetate, propionate and butyrate, respectively. Addition of 10 g inulin-control leads to a 2.0-, 1.6- and 1.4-fold change for daily acetate, propionate and butyrate production, respectively, whereas addition of 10 g inulin-propionate ester leads to a 1.7-, 2.5- and 1.3-fold change for daily acetate, propionate and butyrate production respectively.

### Gastric emptying investigation

Rates of gastric emptying were not significantly different between acute propionate ester and control treatments (Siegel $T_{1/2}$ 185 min [95% CI, 168 to 204] inulin-propionate ester vs. 180 min [95% CI, 163 to 198] control, P=0.506).

### Gut microbial changes to inulin-propionate ester and inulin-control

Inulin control significantly increased Bif164 labelled cells at all time points compared to baseline. Bif164 levels with control were significantly higher compared to the propionate ester cultures throughout fermentation. Both propionate ester and inulin control significantly increased Bac303 labelled cells at all sampling points compared to baseline levels. Ato291 labelled cells increased

significantly with both test substrates between 10 and 34 h compared with baseline concentrations. No other significant changes were seen in any of the groups targeted or total bacteria levels. It appears that propionate ester was fermentable by *Bacteroides* and *Atopobium* but not by *Bifidobacterium* spp.

SUPPLEMENTARY FIGURES

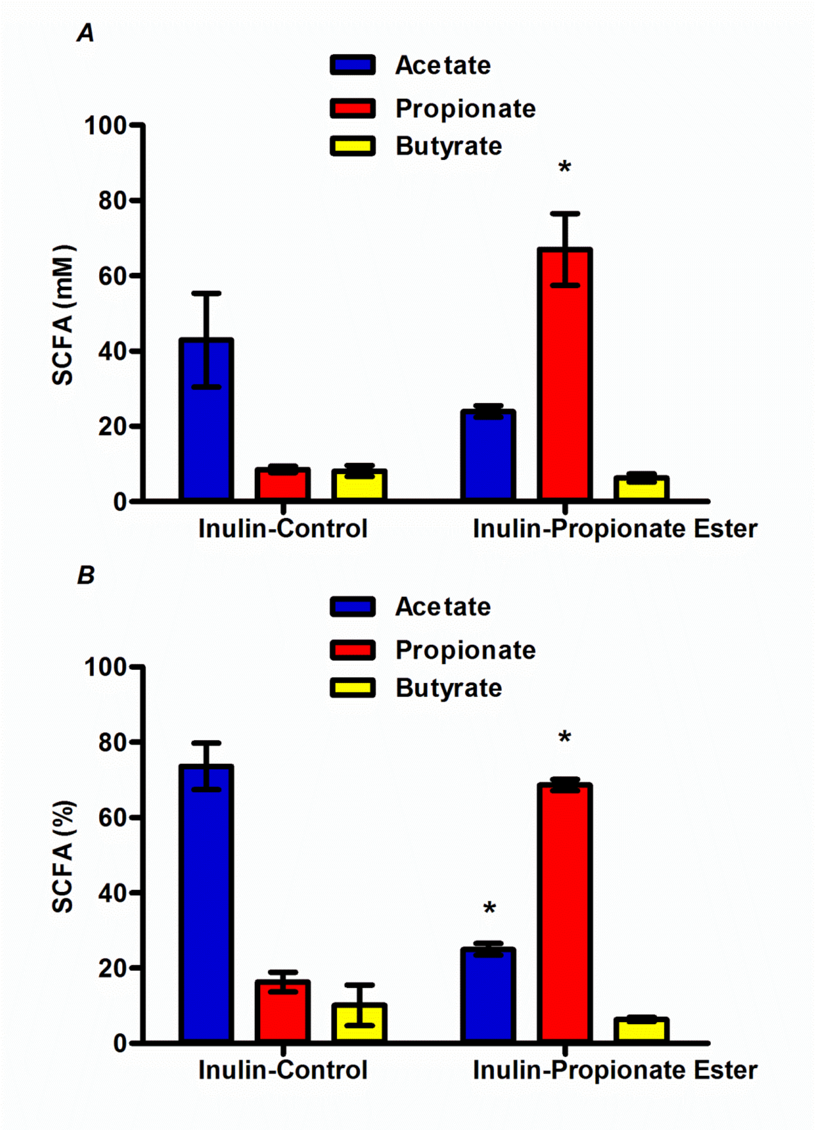


**Suppl. Figure 1. *A.* Short chain fatty acid production and *B.* molar ratios of inulin-control and inulin-propionate ester in fecal fermentations.** Mean ± SEM, *P<0.05 (n=3).

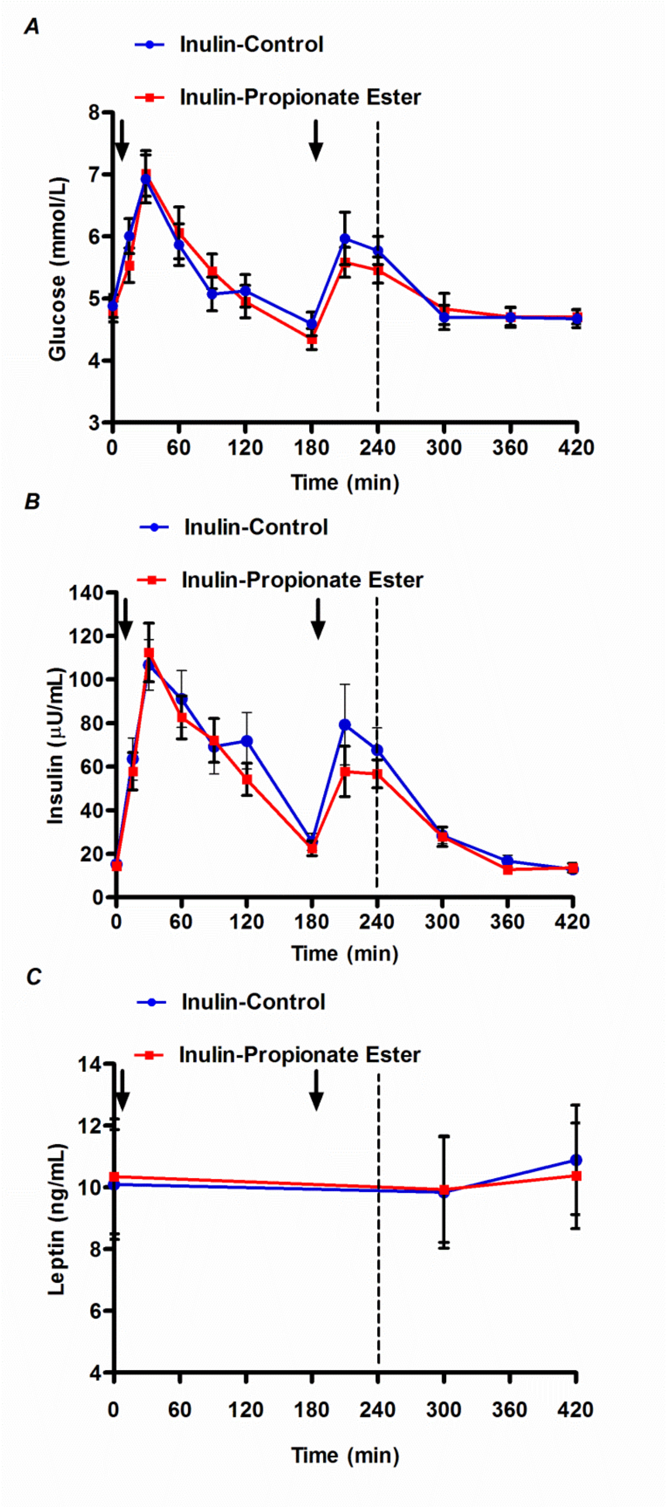


**Suppl. Figure 2. The effect of acute inulin-propionate ester supplementation on postprandial *A*. glucose, *B*. insulin or *C*. leptin response**. Arrows indicate timings of standardized meals. 10 g inulin-control or 10 g inulin-propionate ester were provided with breakfast at 0 min. Dotted lines signify the time point after which >80% inulin-propionate ester enters the colon as determined by the enrichment of $^{13}C$ in expired air and breath $H_2$ methodology (Figure 1C). Data are presented as means ± SEM (n=20).

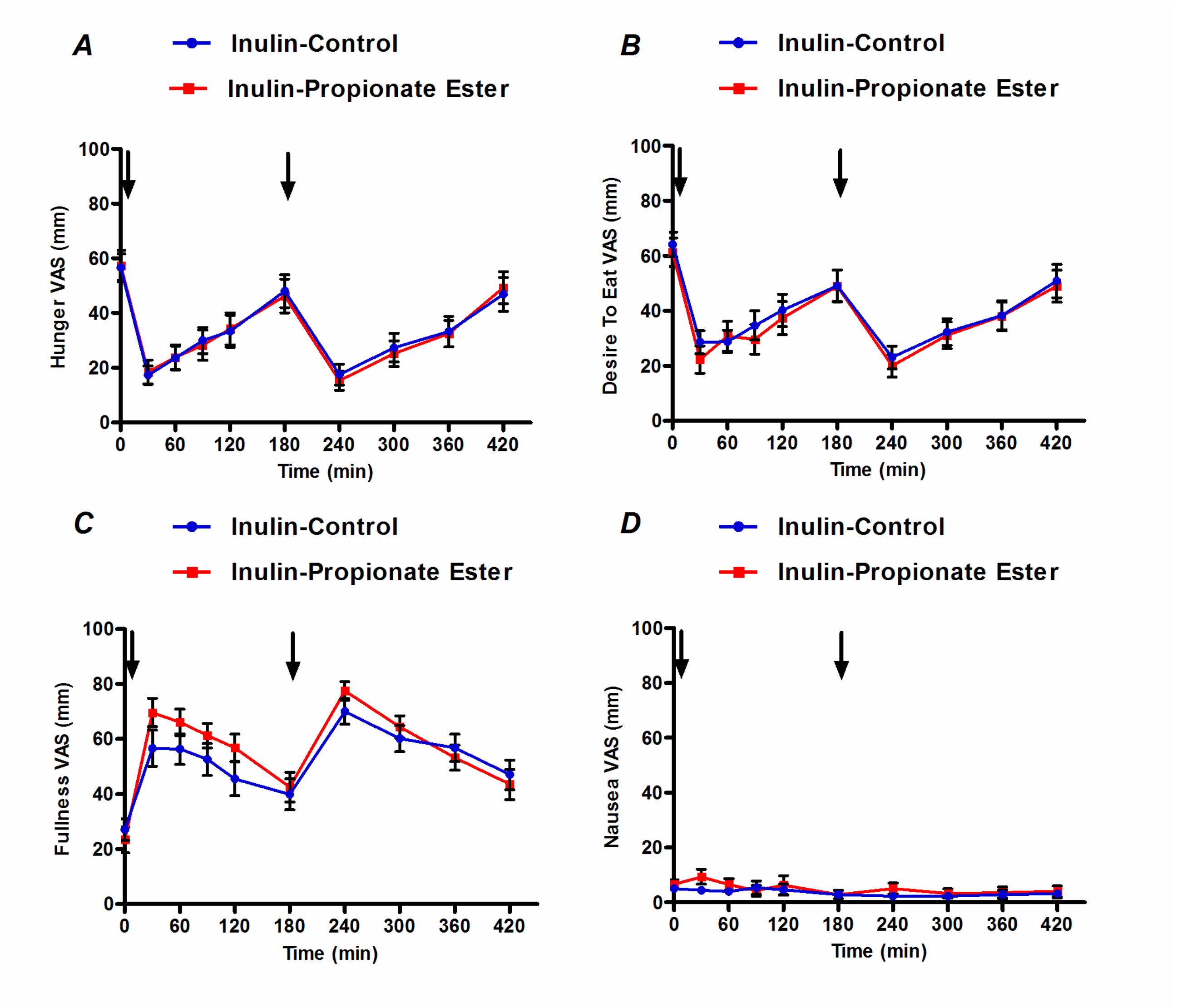


**Suppl. Figure 3. An acute increase in colonic propionate content does not affect postprandial ratings of appetite or nausea**. **A.** *Hunger*, **B.** *Desire To Eat*, **C.** *Fullness*, and **D.** *Nausea*. Ratings were made using 100 mm visual analogue scales (VAS), with extreme statements anchored at each end of the rating scale (e.g. 0 mm *Not at all hungry*, 100 mm *Extremely hungry*). Arrows indicate standardized meals. 10 g inulin-control or 10 g inulin-propionate ester were provided with breakfast at 0 min. Data are presented as means ± SEM (n=20).

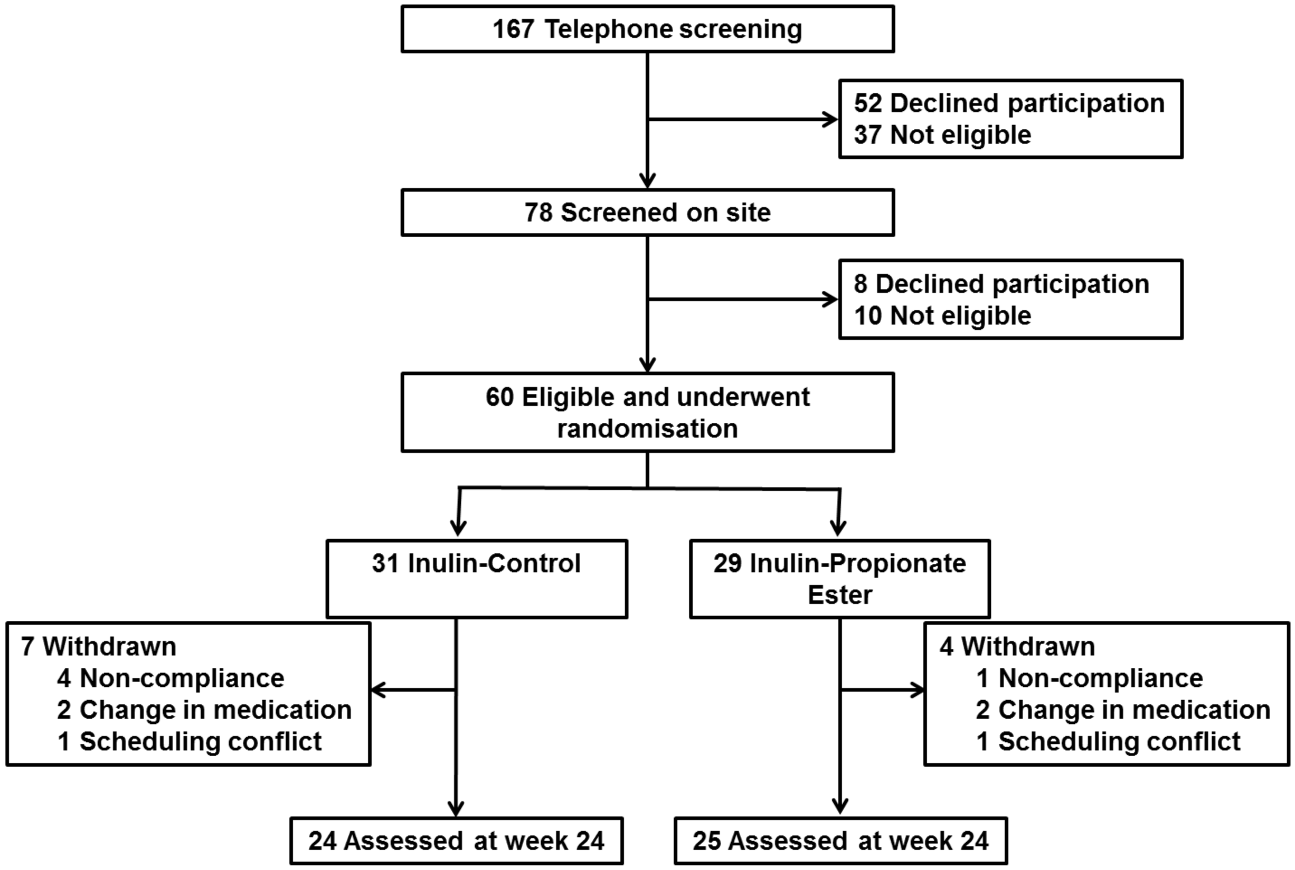


**Suppl. Figure 4. Flow chart showing recruitment and retention in the 24 week supplementation study.**

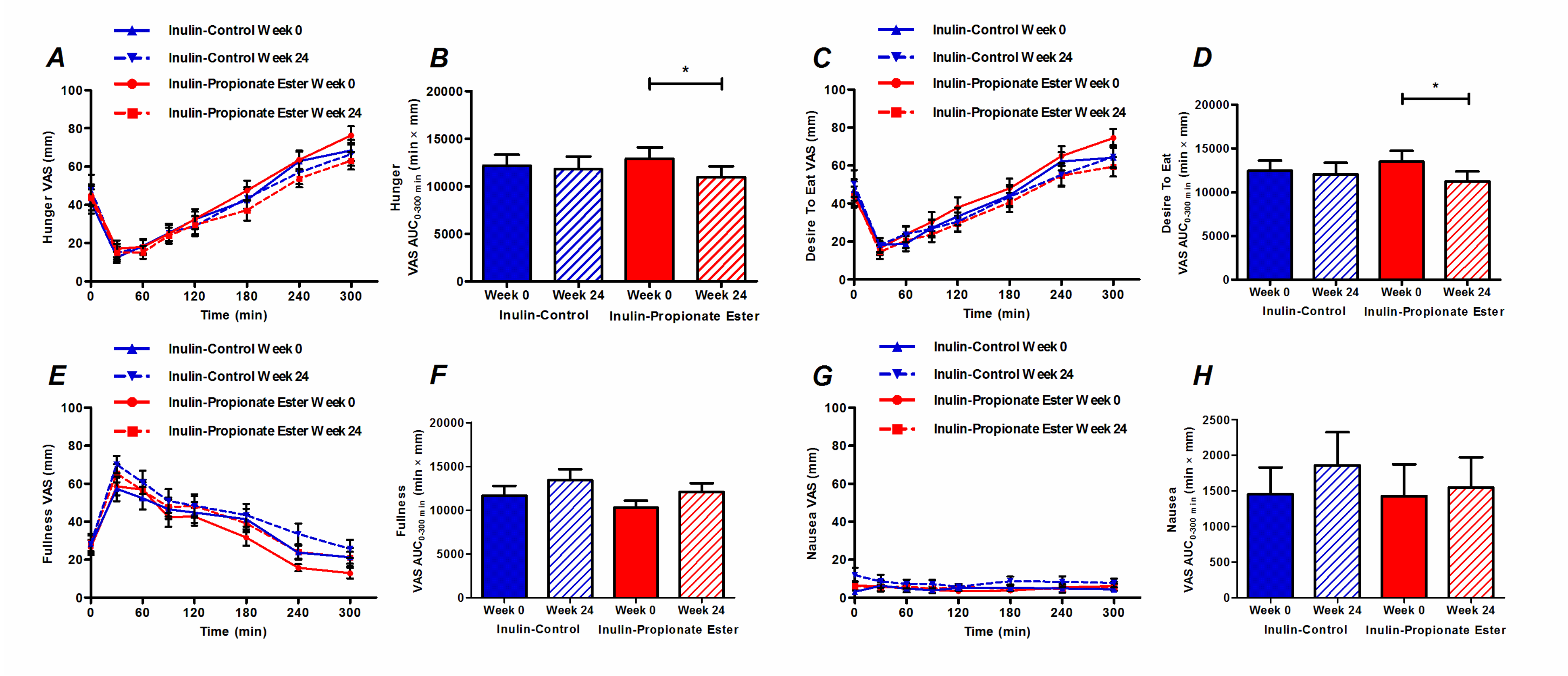


**Suppl. Figure 5. Postprandial ratings of appetite and nausea at baseline and following 24 weeks supplementation with inulin-control and inulin-propionate ester. A.** and **B.** *Hunger*, **C.** and **D.** *Desire To Eat*, **E.** and **F.** *Fullness*, and **G.** and **H.** *Nausea*. Ratings were made using 100 mm visual analogue scales (VAS), with extreme statements anchored at each end of the rating scale (e.g. 0 mm *Not at all hungry*, 100 mm *Extremely hungry*). *P<0.05, Mean ± SEM (inulin-control, n = 24; inulin-propionate ester, n = 25).

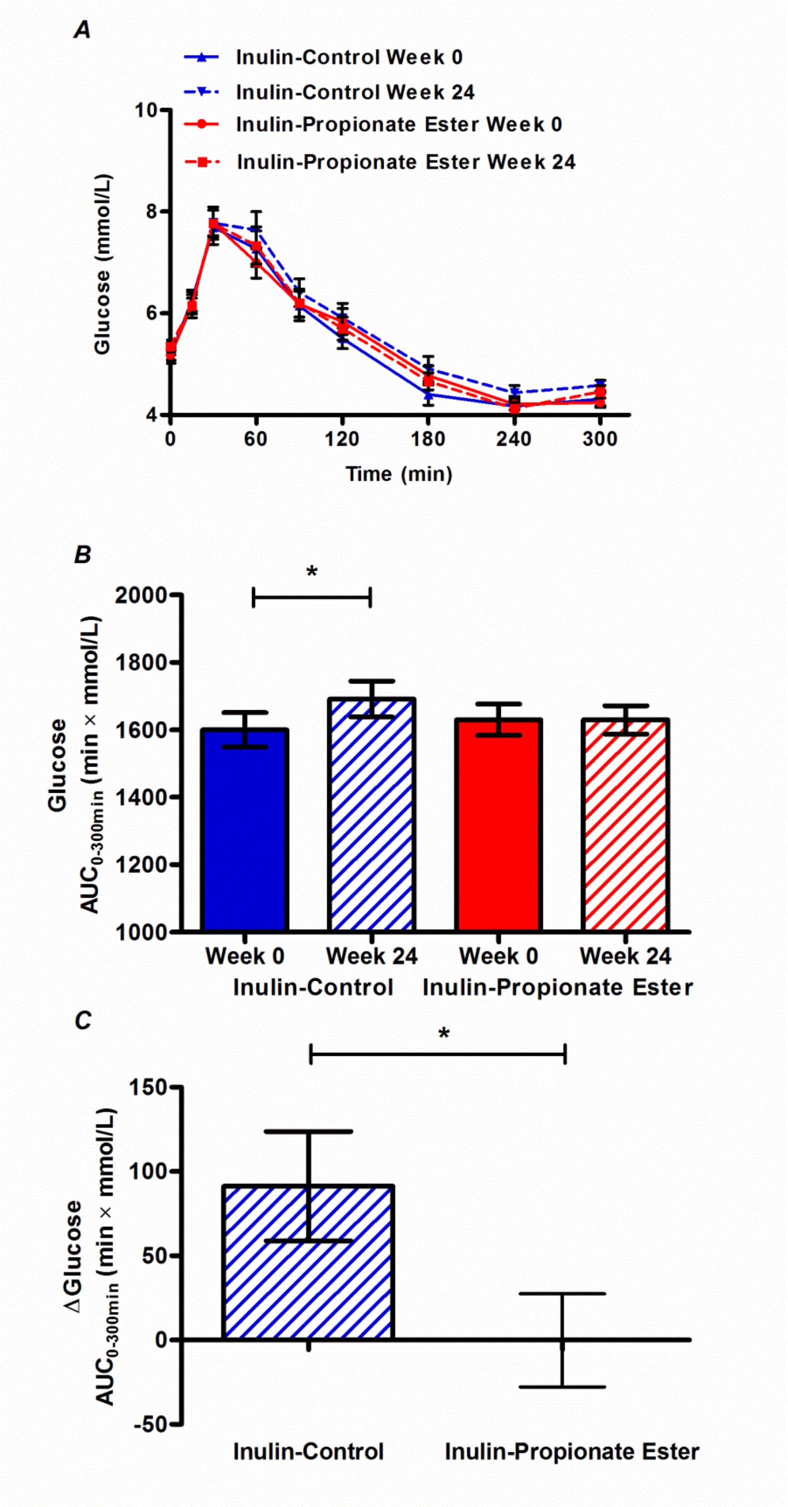


**Suppl. Figure 6. Effect of 24 weeks supplementation with inulin-control and inulin-propionate ester on glucose response. A.** Postprandial glucose response at baseline and following 24 weeks of supplementation with inulin-control and inulin-propionate ester. **B.** Glucose $AUC_{0\text{-}300min}$ at week 0 and week 24. **C.** Change from baseline (week 0) glucose $AUC_{0\text{-}300min}$ at week 24. *$P<0.05$. Mean ± SEM (inulin-control, n = 24; inulin-propionate ester, n = 25).

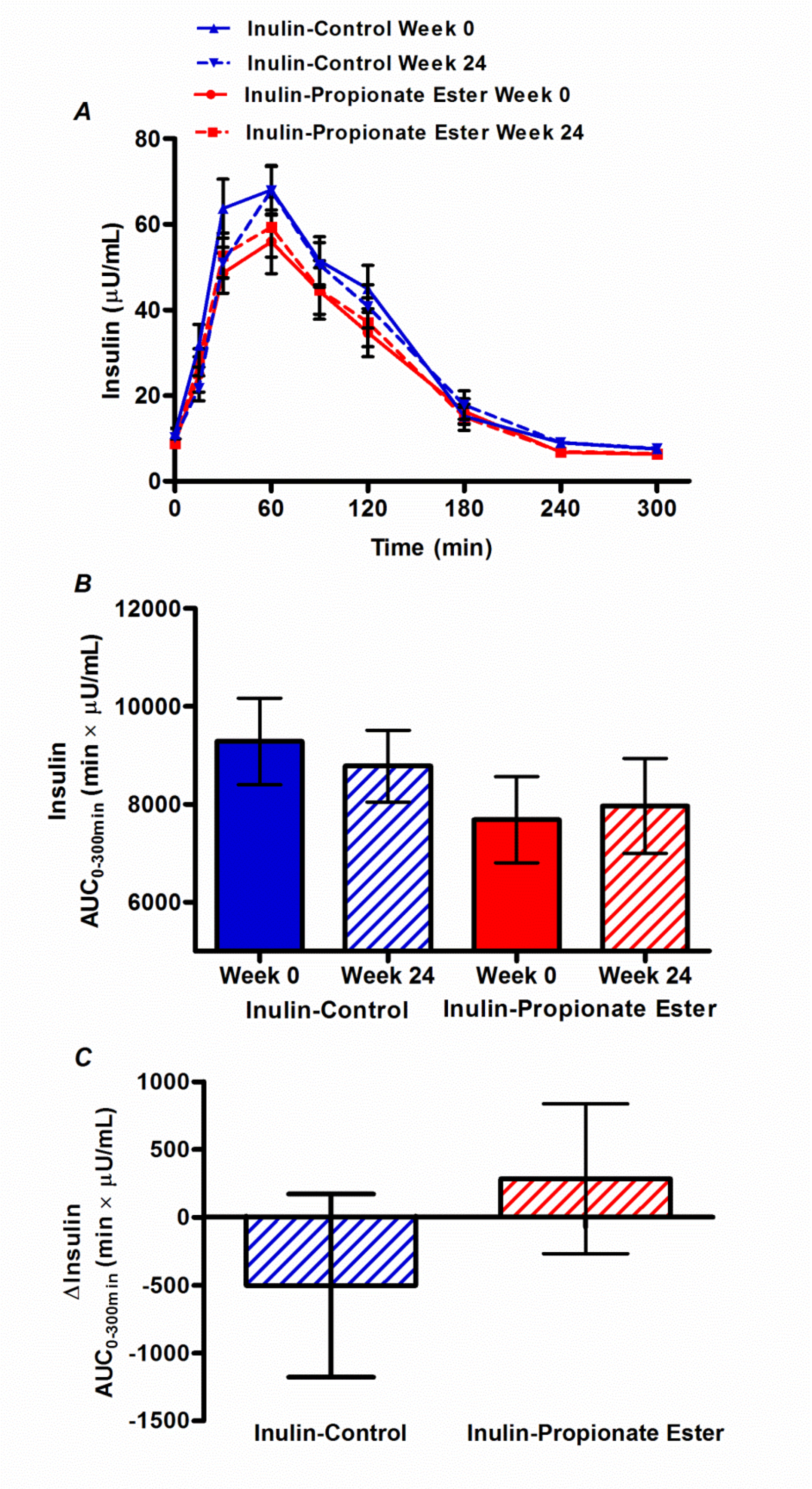


**Suppl. Figure 7. Effect of 24 weeks supplementation with inulin-control and inulin-propionate ester on insulin response. A.** Postprandial insulin response at baseline and following 24 weeks of supplementation with inulin-control and inulin-propionate ester. **B.** Insulin $AUC_{0\text{-}300min}$ at week 0 and week 24. **C.** Change from baseline (week 0) insulin $AUC_{0\text{-}300min}$ at week 24. Mean ± SEM (inulin-control, n = 24; inulin-propionate ester, n = 25).

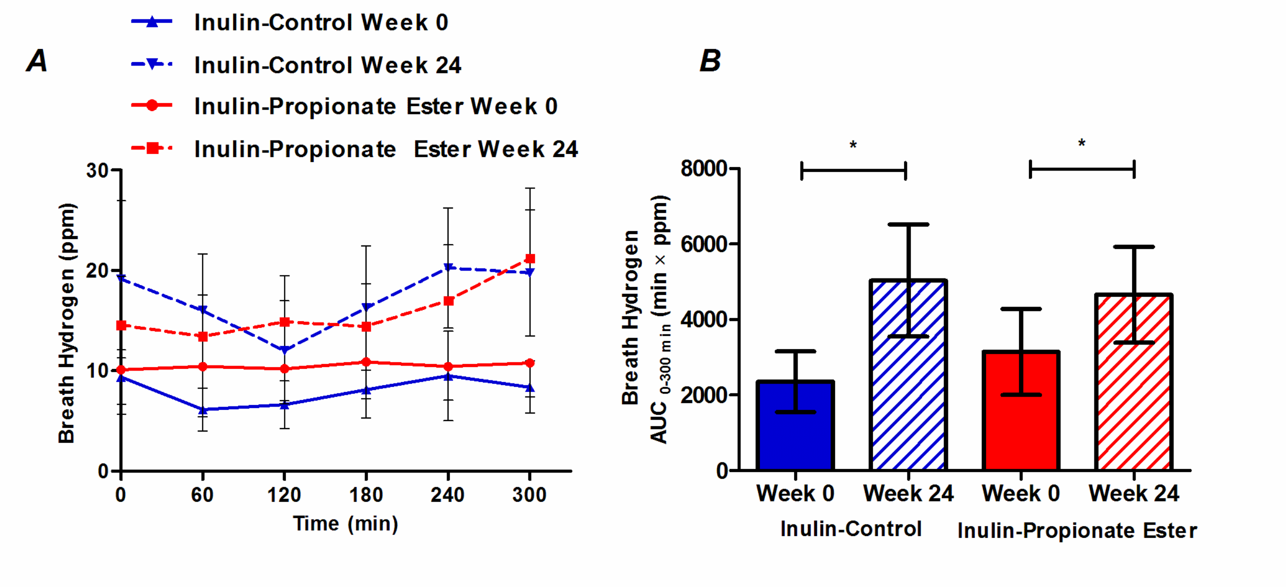


**Suppl. Figure 8. Effect of 24 weeks supplementation with inulin-control and inulin-propionate ester on breath hydrogen.** ***A.*** Fasting and postprandial breath hydrogen at baseline and following 24 weeks of supplementation with inulin-control and inulin-propionate ester and **B.** Breath hydrogen $AUC_{0\text{-}300min}$ at week 0 and week 24. *P<0.05. Mean ± SEM (Propionate Ester, n = 9; Control, n = 8).

## SUPPLEMENTARY TABLES

**Suppl. Table 1. Gastrointestinal adverse events reported during the 24 week supplementation period.**

| | Inulin-Control (N = 24) | Inulin-Propionate Ester (N = 25) | Inulin-Propionate Ester - Inulin-Control | |
|---|---|---|---|---|
| **Side Effect** | **Mean ± SD** | **Mean ± SD** | **Difference (95% CI)** | **P Value** |
| Stomach Discomfort | 31.0 ± 26.6 | 10.5 ± 13.0 | -11.1 (-29.0, 6.7) | 0.221 |
| Nausea | 7.5 ± 10.6 | 4.6 ± 5.8 | 1.5 (-7.4, 10.4) | 0.736 |
| Bloating | 34.0 ± 27.4 | 19.7 ± 22.7 | -6.8 (-24.4, 10.8) | 0.448 |
| Flatulence | 56.0 ± 28.4 | 25.2 ± 22.6 | -24.9 (-41.9, -8.0) | 0.004 |
| Heartburn | 17.8 ± 19.5 | 9.9 ± 14.4 | -1.9 (-18.0, 14.2) | 0.820 |
| Belching | 15.1 ± 14.3 | 10.0 ± 14.0 | -0.0 (-11.8, 11.8) | 1.000 |

Ratings were made at weeks 8, 16 and 24 using 100 mm visual analogue scales (VAS). Subjects were asked to rate the occurrence of each side effect with extreme statements anchored at each end of the rating scale (0 mm *Never*, 100 mm *All the time*).

**Suppl. Table 2. Self-reported food intake and physical activity of subjects at baseline and following 24 weeks of inulin-control and inulin-propionate ester supplementation.** Means ± SEM.

| | Inulin-Control | | Inulin-Propionate Ester | |
|---|---|---|---|---|
| | **Week 0** | **Week 24** | **Week 0** | **Week 24** |
| **Energy (kcal)** | 2215 ± 247 | 2508 ± 317 | 1957 ± 249 | 1753 ± 359 |
| **Energy (kJ)** | 9306 ± 1034 | 10544 ± 1316 | 8213 ± 1043 | 7385 ± 1522 |
| **Protein (g)** | 95 ± 15 | 95 ± 11 | 75 ± 9 | 70 ± 11 |
| **Fat (g)** | 84 ± 12 | 94 ± 18 | 82 ± 13 | 73 ± 12 |
| **Carbohydrate (g)** | 256 ± 19 | 294 ± 43 | 237 ± 29 | 213 ± 68 |
| **Fibre (g)** | 20 ± 4 | 21 ± 5 | 17 ± 2 | 12 ± 5 |
| **Total physical activity (MET-h/week)** | 33.3 ± 8.0 | 31.3 ± 6.8 | 25.7 ± 4.3 | 26.8 ± 4.0 |
| **Vigorous-intensity (MET-h/week)** | 3.5 ± 3.3 | 4.9 ± 3.4 | 4.3 ± 1.8 | 4.4 ± 1.9 |
| **Moderate-intensity (MET-h/week)** | 5.9 ± 2.5 | 4.9 ± 2.5 | 3.6 ± 1.1 | 4.1 ± 0.9 |
| **Walking (MET-h/week)** | 23.9 ± 5.0 | 21.5 ± 3.8 | 17.8 ± 3.7 | 18.3 ± 3.6 |

Energy intake was recorded with 3-day food diaries. Physical activity was measured using the short self-administered format of the International Physical Activity Questionnaire (IPAQ)[13]. IPAQ calculates metabolic equivalents (MET-h/week), derived by assigning standardised MET values for walking, moderate-intensity and vigorous-intensity of 3.3, 4.0 and 8.0, respectively. No significant differences were found in any measure either within- or between-groups (P>0.05).

## REFERENCES


1. Reimann F, Habib AM, Tolhurst G, Parker HE, Rogers GJ, Gribble FM. Glucose sensing in L cells: a primary cell study. *Cell Metab* 2008;8(6):532-9.
2. Habib AM, Richards P, Rogers GJ, Reimann F, Gribble FM. Co-localisation and secretion of glucagon-like peptide 1 and peptide YY from primary cultured human L cells. *Diabetologia* 2013;56(6):1413-6.
3. Cummings JH, Pomare EW, Branch WJ, Naylor CP, Macfarlane GT. Short chain fatty acids in human large intestine, portal, hepatic and venous blood. *Gut* 1987;28(10):1221-7.
4. Sivieri K, Morales ML, Saad SM, Adorno MA, Sakamoto IK, Rossi EA. Prebiotic Effect of Fructooligosaccharide in the Simulator of the Human Intestinal Microbial Ecosystem (SHIME((R)) Model). *J Med Food* 2014;17(8):894-901.
5. Cherbut C, Ferrier L, Roze C, Anini Y, Blottiere H, Lecannu G, et al. Short-chain fatty acids modify colonic motility through nerves and polypeptide YY release in the rat. *Am J Physiol* 1998;275(6 Pt 1):G1415-22.
6. Morrison DJ, Cooper K, Waldron S, Slater C, Weaver LT, Preston T. A streamlined approach to the analysis of volatile fatty acids and its application to the measurement of whole-body flux. *Rapid Commun Mass Spectrom* 2004;18(21):2593-600.
7. Bond JHJ, Levitt MD, Prentiss R. Investigation of small bowel transit time in man utilizing pulmonary hydrogen (H2) measurements. *J Lab Clin Med* 1975;85(4):546-55.
8. Morrison DJ, O'Hara JP, King RF, Preston T. Quantitation of plasma 13C-galactose and 13C-glucose during exercise by liquid chromatography/isotope ratio mass spectrometry. *Rapid Commun Mass Spectrom* 2011;25(17):2484-8.
9. MacDonald AJ, Small AC, Greig CA, Husi H, Ross JA, Stephens NA, et al. A novel oral tracer procedure for measurement of habitual myofibrillar protein synthesis. *Rapid Commun Mass Spectrom* 2013;27(15):1769-77.
10. Verbrugghe A, Hesta M, Gommeren K, Daminet S, Wuyts B, Buyse J, et al. Oligofructose and inulin modulate glucose and amino acid metabolism through propionate production in normal-weight and obese cats. *Br J Nutr* 2009;102(5):694-702.
11. Flint A, Raben A, Blundell JE, Astrup A. Reproducibility, power and validity of visual analogue scales in assessment of appetite sensations in single test meal studies. *Int J Obes Relat Metab Disord* 2000;24(1):38-48.
12. Maes BD, Geypens BJ, Ghoos YF, Hiele MI, Rutgeerts PJ. 13C-Octanoic acid breath test for gastric emptying rate of solids. *Gastroenterology* 1998;114(4):856-9.
13. Craig CL, Marshall AL, Sjostrom M, Bauman AE, Booth ML, Ainsworth BE, et al. International physical activity questionnaire: 12-country reliability and validity. *Med Sci Sports Exerc* 2003;35(8):1381-95.
14. Topping DL, Clifton PM. Short-chain fatty acids and human colonic function: roles of resistant starch and nonstarch polysaccharides. *Physiol Rev* 2001;81(3):1031-64.
